# Human Immune Responses to Plasmodium falciparum infection: Molecular Evidence for a Suboptimal THαβ(TH9) and TH17 Bias over Ideal and Effective Traditional TH1 Immunity

**DOI:** 10.1038/npre.2011.5930.1

**Published:** 2011-04-27

**Authors:** Wan-Chung(Wan-Jiung) Hu, August Louis Bourgeois, Nathan Wolfe, Sher Singh, Anne Jedlicka, Alan Scott

**Affiliations:** 1 Academia Sinica,Taiwan,; 2 PATH, https://www.nature.com/nature; 3 GVFI, https://www.nature.com/nature; 4 National Taiwan Normal University,; 5 Johns Hopkins University School of Public Health,

**Keywords:** microarray, TH9, TH17, Th1, THalpha/beta, malaria, IL-10, interferon gamma, virus, ADCC, NK cell

## Abstract

Using microarray analysis, we showed up-regulation of Toll-like receptors 1,2,4,7,8, NF-[kappa][BETA], TNF-[alpha], p38-MAPK and MHC molecules in human peripheral blood mononuclear cells following infection with Plasmodium falciparum. We report herein further studies based on time-course microarray analysis with a focus on malaria-induced host immunity. Results show that in early malaria; selected immunity-related genes were up-regulated including alpha, beta, and gamma interferon related genes, as well as genes of IL-15, CD36, chemokines (CXCL10, CCL2, S100A8/9, CXCL9, and CXCL11), TRAIL and IgG Fc receptors. During acute febrile malaria, up-regulated genes included alpha, beta, and gamma interferon related genes, IL-8, IL-1[beta], IL-10 downstream genes, TGFB1, oncostatin-M, chemokines, IgG Fc receptors, ADCC signaling, complement-related genes, granzymes, NK cell killer/inhibitory receptors and Fas antigen. During remission, genes for NK receptors, immunoglobins, and granzymes/perforin were up-regulated. When viewed in terms of immunity type, malaria infection appeared to induce a mixed TH1 response, in which α and β interferon driven responses appear to predominate over the more classic IL-12 driven pathway. In addition, TH17 pathway also appears to be playing a significant role in the immunity to Plasmodium falciparum. Gene markers of TH17 (neutrophil-related genes, TGFB1 and IL-6 family (oncostatin-M)) and TH[alpha]/[beta] (IFN-[alpha]/[beta] and NK cytotoxicity and ADCC gene) immunity were up-regulated. Initiation of TH[alpha]/[beta] immune response was associated with an IFN-[alpha]/[beta] response which ultimately resulted in IL-10 and IFN-[gamma] achieved via a different pathway from the more classic IL-12 TH1 pattern. Based on these observations, we speculate that in Plasmodium falciparum infection, TH[alpha]/[beta] and TH17 immunity may predominate over the more traditional TH1 response.

Nature Precedings : hdl:10101/npre.2011.5930.1 : Posted 27 Apr 2011

### Introduction

Host immunity may involve one or a combination of pathways. It has been suggested that TH1 immunity, initiated primarily by IL-12, may play an important immunological role in defense against viruses and intracellular bacteria/parasites, while TH2 responses, driven by IL4, have been shown to be more important against helminthes [1]. TH17 immunity is elicited against extracellular bacteria and this recently described host immunity pathway, is thought to be triggered by TGF-β working in tandem with IL-21 or IL-6 [2]. Dendritic cells are important antigen processing cells and play an important role in stimulating both innate and adaptive immune responses to viruses, fungi and bacteria [3]. In these cells, IL-12 is up-regulated following infection with *E. coli* and *Candida albicans,* but not influenza virus. In contrast, interferon alpha/beta is up-regulated after infection with influenza virus, but not *E. coli* and *Candida albicans*. These observations suggest that IL-12 and IFN-αβ may help drive different immune response maturation pathways [3]**.** It is not clear at this time as to which pathway may predominate in the host response to *Plasmodium falciparum* (*P. falciparum*) infection.

TH1 has been suggested to be the dominant and protective immune response against malaria both in rodents and humans [4-5]. Yet, the blood stage of *P. falciparum* can serve to immunosuppress the host's immune response to the liver stage of the parasite [6]. Dendritic cell maturation is inhibited by *P. falciparum* infected RBCs [7]; monocyte maturation is also suppressed by malaria pigments (hemozoin), and low serum IL-12 was reported in severe malaria [8-9]. These observations suggest that the IL-12 driven TH1 IFNg dominant response patterns seen following *P. falciparum* infection may not be

Nature Precedings : hdl:10101/npre.2011.5930.1 : Posted 27 Apr 2011

functioning optimally; thus, against this background data; one may ask how the infected host is capable of mounting an effective TH1 response against the blood-borne stage of this parasite? An alternative pathway may be needed to achieve this host immune response.

To gain a better understanding of host immune response patterns associated *P. falciparum* infection in humans, we carried out transcriptional profiling using microarray analysis of peripheral blood mononuclear cells (PBMCs) after *P. falciparum* infection. Our earlier analysis showed an up-regulation of gene expression for Toll-like receptor signaling, NF-kB, TNF-α, IFN-γ, IL-1β, p38 MAPK, MHC class I & II molecules [10]. In this study, we further analyzed the data focusing on the specific type or types of host immunity induced by infection. Results indicated an up-regulation of TGF-β and the IL-6 family gene (oncostatin M), both of which induce TH17 immunity. We also observed an elevation in the expression of interferon gamma, NK cell cytotoxicity, antibody-dependent cell cytotoxicity (ADCC), which are triggered by interferon alpha/beta (THαβ), instead of IL-12 (TH1). These results suggest multiple immune pathways may be activated following infection with *P. falciparum*. For the purposed of this thesis we have classified these additional pathways as: THαβ and TH17 immunity. The THαβ pathway is not generally recognized within the immunology community as a distinct T cell maturation pathway while the TH17 response pattern has only recently been recognized [11]. However, THαβ responses in which α and β interferons drive a succession of cellular process leading to mid-moderate IFN-γ production and NK cell and ADCC activation have been described in the host response to viruses but they have not been shown to play an important role in the maturation of the anti-malaria adaptive immune response.

To further clarify this point, traditionally TH1 leads to immunity against intracellular bacteria and protozoa; and THαβ against viral infections. Macrophage activation is characteristic of traditional TH1 immunity, while NK activation (natural cytotoxicity & ADCC) is more characteristic of THαβ immunity. Thus, malaria infection appears to be somewhat unique in that it appears to elicit a combined response reflective of both TH17 and THαβ host immunity.

Nature Precedings : hdl:10101/npre.2011.5930.1 : Posted 27 Apr 2011

### Material and Methods

#### Previous analysis

Subject recruitment, sample collection and preparation, and RNA purification were described previously in Ockenhouse and Hu's paper. Briefly, two groups of subjects were recruited for this study after Johns Hopkins University Institutional Review Board and US Army HSRRB ethical approvals were obtained. In the study cohort, 22 subjects, 20-45 years of age, were recruited from the Walter Reed Army Institute of Research (WRAIR). Subjects agreed to receive mosquito bites from laboratory-reared *Anopheles stephensi* infected by *P. falciparum* (3D7 strain). Once parasitemia was detected in the subjects's peripheral blood, these subjects received treatment with chloroquine. Blood samples were drawn during the un-infected baseline period (U) and again, when parasitemia was found for the first time (Early malaria) (E).

In the other study cohort, 15 adults were recruited from Cameroon, Africa. These subjects were 19-49 years of age with acute *P. falciparum* infection. All suffered from typical relapsing fever and blood smears showed parasitemia. These subjects received at least one week of anti-malarial drug treatment (Cotecxin). Blood drawing was performed during the acute febrile infection period (A) and one month later during the remission period (R). During the remission period, physical exams and blood smears were performed to ensure that malaria symptoms were no longer present and parasitemia was no longer detectable.

Nature Precedings : hdl:10101/npre.2011.5930.1 : Posted 27 Apr 2011

In the first study cohort, PBMCs were separated from whole blood samples by Ficoll-gradient at WRAIR. In the second study cohort, blood was collected in CPT tubes and PBMCs were isolated after centrifuge. A RNA stabilizing reagent-RNA later (Ambion, CA, USA) was added and samples were shipped to the USA on dry ice. Total RNA was extracted from both sets of samples using Trizol. The quality of sample RNA was estimated by spectrometry (OD>1.8) and gel electrophoresis.

**Microarray preparation**

Affymetrix U133A GeneChips (Affymetrix, Santa Clara, CA) were used in this study. The GeneChips contain 22,283 probe-sets, including 14,500 known well-characterized human genes and 18,400 transcripts. Before chip hybridization, a QIAGEN RNeasy cleanup kit was used to purify total RNA. Processing of templates for analysis on the Affymetrix U133A GeneChip was performed in accordance with methods described in the Affymetrix Technical Manual, Revision Three. Total RNA from the blood samples were hybridized into the arrays. Detailed cDNA preparation, in vitro transcription, staining, and scanning of Affymetrix U133A GeneChips were described previously in Chris Ockenhouse and Wanchung Hu's paper.

#### Data analysis

We used GeneSpring software and GeneSpring default normalization to perform one-way ANOVA tests, using un-infected samples as baseline. Significantly up-regulated genes were selected if false discovery rate (FDR) was $<0.05$ and fold change was $>1.5$x when compared to un-infected baseline. These genes were placed into three groups based on expression levels in different stages of the infection. In addition, Pearson's correlation analysis and Rank correlation analysis were performed to find the relationship between expression levels of genes after malaria infection. Pathway analysis was performed by using the Pathway Architect (Statagene Inc.) software to identify the specific immunological pathways involved.

#### Microarray accession numbers

The Affymetrix data sets can be accessed at http://www.ncbi.nlm.nih.gov/geo/ under the accession number GSE5418.

#### Results

A total of 2894 genes were differentially expressed out of the 22,283 probe sets in Affymetrix U133A GeneChip based on our criteria of data analysis. Gene expression patterns were measured in individuals with pre-symptomatic experimental early malarial

Nature Precedings : hdl:10101/npre.2011.5930.1 : Posted 27 Apr 2011

infection and in subjects with naturally-acquired acute febrile malarial infection. A baseline set of data derived from 22 uninfected healthy US subjects was used for gene comparison in samples from early malarial infection, acute febrile malarial infection, and remission stage. This baseline dataset is considered appropriate for comparisons between Cameroonian samples and USA samples because of the difficult in collecting baseline samples from Camerooian subjects that have had no prior exposure to malaria. As indicate previously, the study site in Cameroon is endemic for malaria so it is difficult to find adults in the area that may not already be in the prodromal phase of malaria. The correlation of the average gene expression of baseline samples to the average gene expression from 22 USA subjects and from 15 Cameroonian adults, exceeded 97% indicating comparability of gene expression between datasets collected and processed at the Walter Reed Army Institute of Research for US volunteers and at the Johns Hopkins Bloomberg School of Public Health for Cameroonian subjects.

Nature Precedings : hdl:10101/npre.2011.5930.1 : Posted 27 Apr 2011

**TH1 immunity related gene up-regulation during malaria**

After *P. falciparum* infection, many immune response-related genes of the TH1 immunological pathway were up-regulated (Table 1). Although interferon alpha/beta was not detected in this study, the major transcription factor of interferon alpha/beta synthesis, IRF7, was up-regulated after malaria. Many interferon alpha/beta inducible genes were up-regulated during the early stage of malaria including: tryptophenyl-tRNA (IFI53), interferon alpha inducible protein 27 (IFI27), interferon-induced protein 44 (IFI44), interferon-stimulated protein 15KD (G1P2), interferon regulatory factor 1 (IRF1), 2’-5’-oligoadenylate synthetase 3 (OAS3), signal transducer and activator of transcription 1

(STAT1), interferon stimulated protein 35 (IFI35), Myxovirus resistance 1 (MX1), interferon-induced protein with tetratricopeptide repeat 4 (IFIT4), interferon-induced protein with tetratricopeptide repeat 1 (IFIT1), and interferon-induced protein with tetratricopeptide repeat 2 (IFIT2). The expression levels of the above genes were greater than 2-fold in early malaria, as compared to un-infection baseline. In acute febrile malaria, the expression levels of most of the alpha/beta interferon genes tended to decline and all returned to baseline levels of expression during the remission period (Table 1). Interferon gamma and many interferon gamma-related genes were also up-regulated after malaria. These observations are consistent with a model in which malaria infection induces primarily a TH1 type immune response. These up-regulated genes included: interferon gamma, interferon inducible guanylate binding protein 1 (GBP1), interferon inducible guanylate binding protein 2 (GBP2), Janus kinase1 (JAK1), and transporter with antigen processing 1 (TAP1). The expression levels of GBP1, GBP2, and TAP1 ranged from 10.6 to 2.8 fold above baseline during early stage of malaria, and the expression level of GBP2 was greater than 2-fold change during acute febrile malaria. These interferon gamma inducible genes all returned to baseline levels of expression during the remission periods of the disease. The up-regulation of a number of TH1 chemokines over the malaria disease course was also consistent with a TH1 model of anti-malaria immunity. Up-regulated TH1 related chemokine genes included: CXCL10, CCL3, CCL3 and CCR1. The expression levels of CXCL10 (5.3 fold) was highest during the early phase of malaria, while CCR3, and CCL4 were higher during febrile and recovery stages of the disease (Table 1).

Nature Precedings : hdl:10101/npre.2011.5930.1 : Posted 27 Apr 2011

There were many antibody-dependent cellular cytotoxicity (ADCC) and NK cell-related genes up-regulated after malaria. ADCC-related genes included: Fc receptors (Fc alpha receptor, Fc gamma receptor 3B, Fc gamma receptor 1A, and Fc gamma receptor 3A), and ADCC signaling genes (CD3z, Fyn, DAP12, Syk, PI3K, Rac1, PAK1, MAP2K1, MAP2K2, and Arf6). NK cell related genes included NK cell receptor related genes (KLRC3and KIR3DL2), TNF superfamily ligand 10 (TRAIL), NK cell related transcription factors (CEBPgamma,), interleukin 15, and NK cell restricted granzyme M. Interleukin 15 can enhance NK cell differentiation and proliferation. ADCC is mainly mediated by NK cells, but it can also be mediated by macrophages. Fc receptor and ADCC signaling gene up-regulation is consistent with the ADCC machinery being turned on after *P. falciparum* infection. Among the above genes, gene expression levels were greater than 2-fold change (range 2 to 7 fold; see Table 1) in early malaria including: Fc gamma receptor 1A, Fc gamma receptor 3A, Fc epsilon receptor 1G, and TRAIL. Gene expression levels were greater than 2-fold change in acute febrile malaria including: Fc alpha receptor, Fc gamma receptor 3B, Fc epsilon receptor 1G, PI3K, MAP2K2, Arf6, KLRC3, KIR3DL2, and CEBP gamma (range 2-4 fold). Gene expression levels were greater than 2-fold change in the remission period, including: Fc gamma receptor 3B, Fc epsilon receptor 1G, PI3K, Arf6, KLRC3, and KIR3DL2 range 2-3 fold). Although ADCC signaling genes were not greater than 2-fold, they were higher than 1.5-fold change after malarial infection.

CTL related genes (CD8 T cells in Table 1) were only moderately up-regulated, during malaria  Among these genes, granzyme B, CDC42, and Kras exhibited $\geq$ 2-fold

Nature Precedings : hdl:10101/npre.2011.5930.1 : Posted 27 Apr 2011

change during acute febrile malaria. Granzyme B and Kras were ≥ 2-fold change in the remission period.

Although macrophage/monocyte activation is traditionally considered to be a strong marker for classic TH1 type immunity, we surprisingly found that express levels of most macrophage activation genes were relatively unchanged or slightly suppressed after *P. falciparum* infection (Table 2). These data support the hypothesis that there may be two pathways involved in the induction of TH1 types of immune responses: a THαβ interferon driven path and IL-12 driven path. (See Discussion Section). Most macrophage activation and proliferation related genes were un-changed. (Table 2) IL-12 is clearly the major cytokine needed to initiate a strong classic TH1 type immune response, and IL-12 expression levels remain unchanged over the course of this study (Table 2). This observation suggests that the induction of a TH1-type antimalarial immune response may be driven by cytokines other than IL-12. Like IL-12, other traditional TH1 cytokine: IL-3, M-CSF, and GM-CSF can also cause macrophage proliferation but these gene are also not up-regulated in response to infection with *P. falciparum* (Table2). In contrast, many macrophage inhibition-related genes were up-regulated after malaria including heme oxygenase 1 (HMOX1), JunB, and IRF1. JunB, PU.1 and IRF1 suppress macrophage proliferation. As indicated above, a macrophage differentiation inducer, MafB, is up-regulated after malaria. Its antagonists, JunB and PU.1, were also up-regulated, so the effect of MafB could be suppressed. PU.1 up-regulation can drive monocytes to differentiate into dendritic cells instead of macrophages. Although macrophages can also express Fc receptor genes, we think these Fc receptor expression after malaria should

Nature Precedings : hdl:10101/npre.2011.5930.1 : Posted 27 Apr 2011

mainly occur in NK cells. The Fc receptor signaling pathway (ADCC signaling pathway) in NK cell was all up-regulated (Table 1 ). Also, DAP12 up-regulation can suppress Fc receptor signaling in macrophages, so it is less likely that these up-regulated Fc receptors belong to macrophages. Although IL-10 itself was not up-regulated, many IL-10 downstream genes were up-regulated (Figure 1). IL-10 can activate NK cells and de-activate macrophages. HMOX1 is the major downstream effector molecule to mediate IL-10 inhibition of macrophages. HMOX1 was up-regulated after malaria. HMOX1 generates CO molecule to mediate anti-inflammatory effects in macrophages, which is opposite to iNOS, which generates NO molecule to cause inflammatory effects. Argininesuccinate synthetase (ASS) is the rate-limiting enzyme to synthesize arginine; the substrate of iNOS. iNOS is the central mediator representing macrophage classical activation; and un-changed ASS and iNOS express levels support the notion that macrophages are not being activated after malaria infection. In our study, macrophages were inhibited rather than activated after malaria. In summary, many elements of TH1 immunity were up-regulated following infection with *P. falciparum*, including: interferon alpha/beta inducible genes, interferon gamma inducible genes, NK cell related genes (NK cell cytotoxicity), ADCC related genes, TH1 chemokines, and CD8 T cell related genes. However, macrophages appear to become deactivated and do not appear to proliferate after malarial infection.

**TH17 immunity related gene up-regulation during malaria**

A number of genes associated with the new described TH17 immunity pathway were up-regulated after infection with *P. falciparum* (see Table 3). Of these genes, IL-8

Nature Precedings : hdl:10101/npre.2011.5930.1 : Posted 27 Apr 2011

Nature Precedings : hdl:10101/npre.2011.5930.1 : Posted 27 Apr 2011

showed the greatest degree of up-regulation over the entire course of the disease cycle (early, febrile period and remission periods), with expression levels peaking during the febrile period of illness (8.5 fold increase; see Table 3). Other up-regulated genes included other TH17 related cytokines, IL1β, TGFβ1, and oncostatin M [OSM], as well as TH17 related transcription factors, CEBP delta, and CEBP gamma. In addition, selected neutrophil-related genes were also up-regulated, including: neutrophil attracting chemokines, S100A9, CXCL2, and CXCL3, the neuterophil-related CD molecules, ICAM1, the NADPH oxidases gene NCF1, and acute reaction protein, PGE synthetase2. Many complement related genes were also up-regulated including C3A receptor1, C5 receptor1, Delay-accelerating factor (DAF), and Factor D.

**TH2 immunity related genes unchanged during malaria**

Most TH2 immunity related genes were remarkably unchanged over the course of the malaria disease cycle (Table 4). An extensive analysis of TH2 cytokines and chemokines, as well as Mast cell and eosinophil genes revealed that gene expression level were essentially unaffected by infection with *P. falciparum*; thus providing strong evidence that TH2-type immunity was not significantly initiated during malaria in this study. Expression levels for only a single gene involved in Mast cell activation, Fc epsilon receptor 1A surface receptor, was modified during the malaria disease cycle. This gene was down-regulated during early malaria and mildly up-regulated during remission period.

### Gene-to-gene relationship

We performed Pearson's correlation to analyze gene-to-gene relationship after malaria infection and found a number of relationships in our study subjects that were consistent with observations by other investigators in previous studies. We observed a strong negative Pearson's correlation between the expression levels of STAT1 and STAT4 in early malaria infection and acute febrile malaria (Figure 2, Panel A). There are positive Pearson's correlations between the expression level of STAT1 and Fc receptors(ADCC related genes), but negative correlations between STAT4 and Fc receptors. These observations suggested that STAT1 up-regulated Fc receptors. There is negative correlation between STAT1 and STAT4. Although some previous studies found that STAT4, IL-12R, and Fc receptors are co-expressed, STAT4 is not correlated or even negatively correlated with these up-regulated Fc receptors in malarial infection. Fc receptors suppressed STAT4 expression or STAT4 suppressed Fc receptors(Table 5). Rank correlation has also been done, but there were no significant results. Rank correlation is more strict than Pearson's correlation. If the data is normally distributed, Pearson's correlation analysis is more suitable than Rank correlation analysis.

### Immunological pathways

Pathway analysis was conducted to explore immunological genetic circuitry during malaria infection. We selected IL-4, IL-12, TGFB1/IL-6, IL-17, IFN-$\alpha/\beta$ as the central gene markers to explore possible networking among the 2894 genes under analysis in this study. We were able to identify only a few up-regulated genes in this cluster that centered on IL-4(Figure3-B) or IL-12 (Figure 3-C). In contrast, gene

Nature Precedings : hdl:10101/npre.2011.5930.1 : Posted 27 Apr 2011

networks centered on TGFB1/IL6, IL-17 (Figure 3-D), or IFN-αβ (Figure 3-A) were more dramatically altered during the malaria disease cycle. These results further support a model for malaria immunity, in which, IL-17/IFN-alpha/beta play a more central regulatory role than IL-4 and IL-12. Figure 4 shows a sketch of the major pathways of host immunity that are felt to play a role in recovery from malaria based on the observations made in this study, as well as those made by other investigators in previous research. The changes in their expression levels during the early, acute, and remission stages of *P. falciparum* infection are summarized in Figure 4. As shown in Figure 5, the expression of genes related to TH2 immunity were unchanged following malaria infection; with most changes occurred in genes related to the TH17 and THαβ driven pathway of anti-malaria immunity.

**Validation by plasma and PBMC protein expression**

In our previous analysis, we have found out the concordance between transcription of genes and translation of proteins in malarial infection. We demonstrated the similar up-regulation of ICAM1 level after malarial infection between microarray RNA result and surface protein expression detected by flowcytometry. CXCL10, CXCL9, and CCL2 chemokine expressions are up-regulated in our microarray finding. The above chemokine proteins are also identified in plasma samples after malarial infection. These results show that our microarray analysis is validated. (Figure 1)

Nature Precedings : hdl:10101/npre.2011.5930.1 : Posted 27 Apr 2011

Nature Precedings : hdl:10101/npre.2011.5930.1 : Posted 27 Apr 2011

### Discussion

In previous work, we showed that Toll-like receptor signaling genes, NFkB, TNF-alpha, IFN gamma, IL-1 beta, p38 MAPK, and MHC I & II genes were significantly up-regulated in acute febrile malaria in comparison with un-infected baseline [10]. In this current analysis, we identified more immunology genes, whose expression levels were significantly changed at different time-points over the course of the malaria disease cycle. Based on these results, a clearer molecular picture of the range of host immune responses that develop following *P. falciparum* has started to emerge, which suggests that the THαβ variant pathway of original TH1 immunity and TH17 pathway may predominate over the more traditional IL-12 driven TH1 immunity pathway. However, it is important to remain somewhat cautions in the interpretation of the results since the health status of the patients from Cameroon and their genetic background are likely to be different from those of US volunteers. It is also important to know that PBMCs being analyzed are mix cell populations. Sub-populations of cell of PBMC preparation include cells, CD4 T cells, CD8 T cells, NK cells, and monocytes. This cellular heterogeneity could have significant impact on the gene expression profiles seen after malaria infection. Because the RNA-later reagent used to prevent RNA degradation in the PBMC preparation process , interferes with the surface markers of PBMCs, we were not able to carry out cell flow cytometry to isolate individual cell fractions for analysis. Consequently, further studies are needed to know the gene expression profiles of individual lymphoid cell populations after acute malaria infection. Hopefully the data presented in this thesis will help to direct this future research.

.

Basically, host immune response can be categorized into TH1, TH2, and TH17 immune response pathways. TH1 immune responses (Type 1 immunity) are IFN-γ and IgG mediated and they play an important role in host responses to viruses and intracellular bacteria/protozoa, TH2 immune responses are IL-4 and IgE mediated immunity that are important to host responses to helminthes, and TH17, initiated by TGF-β, IL-6 and IL-17 and they are important in host responses to extracellular bacterial pathogens [1-2,12]. In this research, our data suggests that there should be a new immunological pathway derived from the original TH1 immune response: THαβ. THαβ is named after IFNα/β. In this model, THαβ is triggered by interferon alpha/beta ±IL-10 and TH1 is triggered by interleukin 12. Based on previous work by others, THαβ is important in host immunity against virus infection; TH1 is important in host immunity against intracellular bacteria/protozoa. Different types of host immunity have different effector cells. In TH2 immunity, the effector cells are *mast cells*, *eosinophils, basophils*, IgE/IgG4 secreting B cells, and IL-4 secreting CD4 T cells. In TH17 immunity, the effector cells are *neutrophils*, IgA/IgM/IgG2 secreting B cells, and IL-17 secreting CD4 T cells. In THαβ immunity, the effector cells are *NK cells*, IgG1 secreting B cells, CTL, and IL-10(+++)/IFN-γ(+) secreting CD4 T cells. In TH1 immunity, the effector cells are *macrophages*, IgG3 secreting B cells, CTL, and IFN-γ(+++) secreting CD4 T cells. Similarly different types of host immunity use different STAT proteins to initiate specific immune reactions. IL-4 activates STAT6 in TH2 immunity. TGF-β and IL-6 activates STAT3β in TH17 immunity. Interferon alpha/beta and IL-10 activates STAT1 and STAT3α in THαβ immunity. IL-12 activates STAT4 in TH1 immunity [13]**.** TGF-β and IL-2 activate STAT5 in Treg immunity. Based on our data, malaria infection appears to

Nature Precedings : hdl:10101/npre.2011.5930.1 : Posted 27 Apr 2011

induce a mixed TH17 and THαβ immune responses which best fits with a model in which both pathways are activated during the onset and recovery from malaria infection.

Others have shown that TGF-β with IL-6 can induce TH17 immunity [2,12]. TH17 lymphocytes also produce large amounts of IL-8, TNF-α and IL-1 which recruit and activate neutrophils. Due to our previous analysis, Toll-like receptor 1,2,4,7,8 are up-regulated after malarial infection. TLR1/2/4 mainly induce anti-extracellular bacteria TH17 immunity. TLR7/8 binding to single strand RNA mainly induce ant-virus THαβ immunity. In addition, we found a lot of heat shock proteins are up-regulated after malarial infection. Heat shock proteins, mainly HSP60 or HSP70, can activate TLR2/4 for inducing TH17 immunity. In this study, TGF-β, IL-1, and IL-8 were strongly up-regulated in acute malaria (Table 3). Although IL-17 was not up-regulated, the IL-6-related cytokine family gene-Oncostatin M was up-regulated in acute malaria infection. Oncostatin M can up-regulate STAT3β, a key mediator in TH17 immunity [14]. Thus, it appears that TH17 immunity can be induced in malaria infection. Up-regulated TGF-β, IL-6, or TNFα are associated with a higher frequency of malaria complications and an overall poor malaria prognosis. A previous study revealed that neutrophil-related genes were up-regulated in children after acute *P. falciparum* malaria infection [15]. They also reported up-regulation of IL-6 receptor, C3AR1, C5R1, FPRL1, PBEF, and IL-1β which are important components of TH17 immunity. Neutrophilia has been observed in falciparum malaria, and elevated WBC counts are associated with severe malaria [16]. Gene network analysis of TGFB1/IL-6 or IL-17 related gene expression after malaria infection also suggested that malaria was TH17 dependent. *Falciparum* malaria often causes

Nature Precedings : hdl:10101/npre.2011.5930.1 : Posted 27 Apr 2011

complications such as acute renal failure and acute respiratory distress syndrome (ARDS). Neutrophil overactivation in TH17 immunity is thought to play major role in the pathogenesis of cerebral malaria, acute renal failure, and ARDS [17-19]. Thus, malaria induced TH17 immunity could be related to both malaria-induced cerebral malaria, acute renal failure, and ARDS.

Evidence for dividing the more traditional TH1 immunity pathway into two subtypes (THαβ and TH1 immunity) comes primarily from both mouse and human models of malaria immunity. The major difference separating TH1 immunity and THαβ immunity are the effector cells involved. In the mouse model, interferon alpha/beta suppresses macrophage proliferation and neutrophiles and NK cells play a more prominent role, in contrast, IL-12 enhances macrophage proliferation [20-21]. In addition, interferon alpha/beta increases NK cell blastogenesis, so alpha/beta interferon enhances NK cell proliferation [22]. Thus, interferon alpha/beta and IL-12 mediated immunological events can be distinguished by the different effector cells they enhance, NK cells or macrophages.

Both IL-12 and interferon alpha/beta can induce CD4 T cells to secret interferon gamma. Interferon alpha/beta can induce CD4 T cells to produce both interferon gamma(+) and IL-10(+++). These interferon gamma and IL-10 secreting CD4 T cells were previously called a type 1 regulatory T cells because IL-10 has some anti-inflammatory effect. However, if these types of T cells are purely immuno-regulator cells, it cannot explain why there is interferon gamma production from these CD4 T cells. IL-

Nature Precedings : hdl:10101/npre.2011.5930.1 : Posted 27 Apr 2011

10 is not a pure immunosuppressant because it can stimulate NK cells, B cells and CTLs. In fact, the regulatory effect of these Tr1 cells could be due to cross-regulation between Tr1 cells and other T helper cells such as TH1, TH2, and TH17 cells. In a prior mouse study, interferon alpha/beta can substitute for IL-12 in the induction of interferon gamma production associated with the development of TH1 immunity [23]. In mice, interferon alpha/beta can also induce B cell isotype switching to produce IgG antibody [24]. IL-10 can stimulate B cell to produce IgG1 antibody and IFNg can stimulate B cells to produce IgG3 antibody. Interferon alpha/beta can also cause cross-priming of CD8 T cells [25]. Both interferon alpha/beta and IL-12 can serve as a third signal to facilitate clonal expansion of antigen specific CD8 T cells [26]. Again in mice, interferon alpha/beta can suppress the proliferation of IL-17 secreting CD4 T cells [2]. These observations in mice strongly suggest that interferon alpha/beta can induce an alternative TH1 immunity without the need of IL-12. In addition, interferon alpha/beta suppresses the induction of nitric oxide synthetase (iNOS) [27]. iNOS is the central effector molecule involved in the killing of ingested bacteria and parasites by macrophages. Activation of iNOS is a basic tenet of classic macrophage activation. iNOS activation has been shown to enhance the intracellular killing capability of macrophages by a 1000-fold [28]. iNOS can also be induced by interferon gamma through IL-12 mediated up-regulation. IL-10 can also serve as a strong macrophage de-activator. It can suppress macrophage proliferation, iNOS activation, and macrophage cytokine production. It can also suppress IL-12 production. Thus, interferon alpha/beta and IL-12 should be seen as belonging to different inductive immunological pathways.

Nature Precedings : hdl:10101/npre.2011.5930.1 : Posted 27 Apr 2011

Mouse models of anti-malaria immunity indicate that interferon gamma and TH1 immunity play central roles in the developmental process [5]. Although administration of IL-12 can provide 100% protection against malaria parasite challenge, IL-12 plays a limited role in natural immunity against malaria in mice [4]. Active immunosuppression is well documented during malaria infection in mice [6]. IL-12 expression is down relegated in this model [29]. According to an IRF1 knockout study, mice can initiate TH1 immunity by bypassing the need for IL-12 production through the use of an alternate interferon alpha/beta driven pathway [30]. Based on recent microarray studies in malaria infected mice there is no evidence of IL-12 up-regulation following infection. Instead, interferon alpha/beta and its related genes were found to be significantly up-regulated [31-32]. Thus, providing further evidence at the gene level that interferon alpha/beta can substitute for IL-12 in the induction of malaria-specific interferon gamma TH1 type immune response after infection in rodents. However, the IFNαβ driven THαβ immunity only can produce mid-to-moderate IFNg, and IL-10, the main effector molecule in THαβ immunity, antagonizes the effect of IFNg upon macrophages. Because the ideal traditional TH1 immunity cannot be triggered in natural malarial infection in human to clear out the protozoa, malaria parasites can cause severe illness in human.

In human, there is also a substantial amount of evidence indicating that TH1 immunity can be induced by two pathways driven by αβ interferon or IL-12 [33]. Administration of IL-12 to human subjects suppresses NK cell proliferation [34]. Macrophage activation serves to inhibit NK cell function; while NK cell activation serves to inhibit macrophage proliferation [20,35]. Given these observation it is reasonable to

Nature Precedings : hdl:10101/npre.2011.5930.1 : Posted 27 Apr 2011

speculate that these two effector cells belong to different immunological pathways.In the present study, we found that a number of genes involved in the NK cell proliferation were up-regulated over the course of the malaria disease cycle (Table 1). In addition, as in the mouse model, genes associated with macrophage proliferation were not up-regulated following infection with *P. falciparum*. Increased NK cell populations have also been reported previously in acute malaria infection [36-37]. Thus, it appears that the interferon alpha/beta-NK cell pathway of THαβ immunity is preferentially enhanced after *P. falciparum* infection; while the IL-12 driven pathway appears unaffected. Other THαβ related cytokines also can cause NK cell activation or proliferation including IL-10 and IL-15, respectively.

In human, interferon alpha/beta has been shown to enhance NK cell cytotoxicity as well as antibody dependent cell cytotoxicity (ADCC) [38], but IL-12 administration suppresses NK cell cytotoxicity and ADCC [39]. In addition, IL-12 induces NK cell to secret high levels of interferon gamma to activate macrophages instead of activating NK cell's cytotoxicity machinary [22]. In the current study, Fc receptors, alpha/beta interferon and ADCC related genes were up-regulated after malaria infection. Consistent with this model, patients with defects in NK cell production usually suffering from recurrent virus infections and have more complications following malaria infection [40 41].

Further evidence that interferon alpha/beta can trigger TH1 immunity without the need for IL-12 comes from other human studies, in which, interferon alpha/beta suppressed STAT6 expression which is related to IL-4 induction and expression of TH2

Nature Precedings : hdl:10101/npre.2011.5930.1 : Posted 27 Apr 2011

immunity [42]. In our study interferon alpha/beta inducible genes were clearly up-regulated to induce THαβ immunity with ADCC; while TH2 related gene expression remained unchanged. In human models, interferon alpha/beta inhibits IL-12 production via an IL-10 dependent mechanism[43]. Thus, it seems reasonable to speculate that IL-12 and interferon alpha/beta do not belong to the same immunological pathway.

In human malarial infection, it has been shown that serum IL-12 levels are inversely correlated with malaria parasitemia [9]. The more severe the malaria is, the lower IL-12 expression levels become. The up-take of malarial pigment (Hemozoin) can down-regulate IL-12 secretion in human monocytes [8]. Malarial PGE2-like molecules can also down-regulate IL-12 secretion [44]. Up-regulation of CD36 by malarial infection can inhibit dendritic cell maturation with decreasing IL-12/IL-10 ratio [7]. Although hemozoin, in certain studies, can activate TLR9 which is for traditional TH1 immunity, TLR9 is not detected in our study. In previous microarray analysis of human malarial infection, interferon inducible genes were up-regulated with un-changed IL-12 expression [15,45]. Other important components of THαβ immunity included up-regulation of HMOX1, FAS, BCL6, TNFRSF10A, and MIP1α were noted in Griffiths'research which studied the peripheral blood leukocytes from Kenyan children with acute falciparum malaria. In this microarray study, we found up-regulation of interferon alpha/beta, interferon gamma, NK cell activating receptors, Fas, TRAIL, Fc gamma receptors, ADCC related genes, IL-15, granzymes, and TH1 related chemokines (IP10, CXCL9, CXCL11, CCL3, CCL4, CCR1). These observations are, in general, consistent with the development of THαβ immunity characterized by NK cell activation induced by alpha/beta interferon following

Nature Precedings : hdl:10101/npre.2011.5930.1 : Posted 27 Apr 2011

Nature Precedings : hdl:10101/npre.2011.5930.1 : Posted 27 Apr 2011

*P. falciparum* infection. Genes associated with TH1 immunity driven by IL-12 and macrophage activation (iNOS up-regulation) were un-changed after malarial infection. In fact, genes coding for a number of macrophage deactivation molecules were up-regulated including TGFβ receptor, CD36, CD163-Heme oxygenase1, and CD47-SIRPα. These molecules can actively suppress macrophage activation. When given alone and prior to malaria infection IL-12 can provide 100% protection against experimental *Plasmodium* infection through the activation of macrophages, which help to control the liver stage of infection [4]. However, based on the data collected during the course of the current study it also appears that infected individuals defend malaria through an alternate THαβ immunity, involving alpha/beta interferon and the activation of NK cells. Interferon gamma plays a central role in immunity against malaria. However, the natural host immunity is suboptimal because IFNg is not the main effector in THαβ immunity. The major effector cells of THαβ immunity are NK cells, and the major effector cells of traditional TH1 immunity are macrophages. NK cells kill virus-infected cells via ADCC machinery. During NK ADCC, granzyme released from NK cells into target cells will initiate cell apoptosis process. During this cellular apoptosis, DNA and RNA fragmentation will occur to chop out all the integrated form virus DNAs or latent form virus DNAs/RNAs by activated DNase/RNase in host genome. Thus, virus will be killed via this NK cell ADCC. Malaria protozoa live in RBCs which are mainly digested by macrophages. Intracellular protozoa and bacteria are mainly destroyed by phagolysosome with iNOS, $H_2O_2$, and lysozyme activation inside macrophages. It is worth noting that interferon gamma significantly up-regulates macrophage phagocytosis ability. Thus, macrophage activation and phagocytosis is key in the host defense against intracellular bacteria/protozoa infection. That is why traditional TH1 immunity is the ideal and most effective immunity against malarial protozoa.

Although IL-10, a strong macrophage de-activator, was not up-regulated in this study, many downstream genes of IL-10 were up-regulated including heme oxygenase 1, and it suggests that IL-10 is actually up-regulated after malaria infection. In an ideal situation, TH1 immunity is basically immunity against intracellular bacteria/protozoa; THαβ immunity is basically immunity against viruses. Although, it appears that macrophages may not be activated in natural immunity against malaria, interferon gamma(+) and IL-10(+++) secreting CD4 T cells, cytotoxic CD8 T cells, IgG secreting B cells, and NK cells are up-regulated by interferon alpha/beta in malarial infection. In a clinical study in Gabon, interferon-γ and IL-10 secreting CD4 T cells were increasing and correspondent to the recovery of *P. falciparum* infection [46]. Pure TH1 interferon-γ producing T cells and pure TH2 IL-4 producing T cells were not changed during convalescence after malaria infection. Thus observation is consistent with a model, in which, interferon-γ and IL-10 secreting T cells play important roles in host immunity against malaria. In conclusion, it appears that the human immune response to *P. falciparum* is characterized by a suboptimal THα/β and TH17 bias which predominates over the more ideal and effective traditional TH1 responses driven by IL-12. Since treatment for malarial infection faces some difficulty like drug resistance, we strongly encourage to use IFNg(FDA approval drug) or IL-12 for malarial infection treatment to achieve the optimal host immunity to kill the intracellular protozoa.

Nature Precedings : hdl:10101/npre.2011.5930.1 : Posted 27 Apr 2011

Nature Precedings : hdl:10101/npre.2011.5930.1 : Posted 27 Apr 2011

**Acknowledgements**

We thank Dr. Roger Moyou for his help of accessing patients in Cameroon. We thank M. Nau and A. Arnold for expert technical assistance.

This work was supported by two different grants. The first grant is from the Malaria Research Institute of Johns Hopkins University. The second grant is provided by Cooperative Agreement number W81XWH-04-2-2005 between the U.S. Army Medical Research and Material Command and the Henry M. Jackson Foundation for the Advancement of Military Medicine and by the U.S. Army Medical Research and Material Command. Additional support was provided by the U.S. Embassy Cameroon, the JHU Cameroon Program, and the government of Cameroon.

This study is part of Wan-Chung Hu (Wan-Jiung Hu)'s PhD thesis in Johns Hopkins University in 2007 August. In 2008, a subsequent paper called the new immunological pathway, TH9 immunity, for the IL-10/IL-9 secreting CD4 T cells. However, the authors here made the discovery earlier. TH9 immunity should be renamed as THαβ immunity.

## References

1 Mosmann, T. R., Cherwinski, H., Bond, M. W., Giedlin, M. A. & Coffman, R. L. Two types of murine helper T cell clone. I. Definition according to profiles of lymphokine activities and secreted proteins. *J Immunol* **136**, 2348-2357 (1986).

2 Harrington, L. E. *et al.* Interleukin 17-producing CD4+ effector T cells develop via a lineage distinct from the T helper type 1 and 2 lineages. *Nat Immunol* **6**, 1123-1132 (2005).

3 Huang, Q. *et al.* The plasticity of dendritic cell responses to pathogens and their components. *Science* **294**, 870-875 (2001).

4 Sedegah, M., Finkelman, F. & Hoffman, S. L. Interleukin 12 induction of interferon gamma-dependent protection against malaria. *Proc Natl Acad Sci U S A* **91**, 10700-10702 (1994).

5 Su, Z. & Stevenson, M. M. Central role of endogenous gamma interferon in protective immunity against blood-stage Plasmodium chabaudi AS infection. *Infect Immun* **68**, 4399-4406 (2000).

6 Ocana-Morgner, C., Mota, M. M. & Rodriguez, A. Malaria blood stage suppression of liver stage immunity by dendritic cells. *J Exp Med* **197**, 143-151 (2003).

7 Urban, B. C. *et al.* Plasmodium falciparum-infected erythrocytes modulate the maturation of dendritic cells. *Nature* **400**, 73-77 (1999).

8 Keller, C. C. *et al.* Acquisition of hemozoin by monocytes down-regulates interleukin-12 p40 (IL-12p40) transcripts and circulating IL-12p70 through an IL-10-dependent mechanism: in vivo and in vitro findings in severe malarial anemia. *Infect Immun* **74**, 5249-5260 (2006).

9 Boutlis, C. S. *et al.* Plasma interleukin-12 in malaria-tolerant papua new guineans: inverse correlation with Plasmodium falciparum parasitemia and peripheral blood mononuclear cell nitric oxide synthase activity. *Infect Immun* **71**, 6354-6357 (2003).

10 Ockenhouse, C. F. *et al.* Common and divergent immune response signaling pathways discovered in peripheral blood mononuclear cell gene expression patterns in presymptomatic and clinically apparent malaria. *Infect Immun* **74**, 5561-5573 (2006).

11 Park, H. *et al.* A distinct lineage of CD4 T cells regulates tissue inflammation by producing interleukin 17. *Nat Immunol* **6**, 1133-1141 (2005).

12 Bettelli, E. *et al.* Reciprocal developmental pathways for the generation of pathogenic effector TH17 and regulatory T cells. *Nature* **441**, 235-238 (2006).

13 Yu, C. R. *et al.* Differential utilization of Janus kinase-signal transducer activator of transcription signaling pathways in the stimulation of human natural killer cells by IL-2, IL-12, and IFN-alpha. *J Immunol* **157**, 126-137 (1996).

14 Zhang, F., Li, C., Halfter, H. & Liu, J. Delineating an oncostatin M-activated STAT3 signaling pathway that coordinates the expression of genes involved in cell cycle regulation and extracellular matrix deposition of MCF-7 cells. *Oncogene* **22**, 894-905 (2003).

Nature Precedings : hdl:10101/npre.2011.5930.1 : Posted 27 Apr 2011

15 Griffiths, M. J. *et al.* Genomewide analysis of the host response to malaria in Kenyan children. *J Infect Dis* **191**, 1599-1611 (2005).
16 Ladhani, S., Lowe, B., Cole, A. O., Kowuondo, K. & Newton, C. R. Changes in white blood cells and platelets in children with falciparum malaria: relationship to disease outcome. *Br J Haematol* **119**, 839-847 (2002).
17 Senaldi, G., Vesin, C., Chang, R., Grau, G. E. & Piguet, P. F. Role of polymorphonuclear neutrophil leukocytes and their integrin CD11a (LFA-1) in the pathogenesis of severe murine malaria. *Infect Immun* **62**, 1144-1149 (1994).
18 Pittet, J. F. *et al.* TGF-beta is a critical mediator of acute lung injury. *J Clin Invest* **107**, 1537-1544 (2001).
19 Schrier, R. W., Wang, W., Poole, B. & Mitra, A. Acute renal failure: definitions, diagnosis, pathogenesis, and therapy. *J Clin Invest* **114**, 5-14 (2004).
20 Bordignon, C. *et al.* Inhibition of natural killer activity by human bronchoalveolar macrophages. *J Immunol* **129**, 587-591 (1982).
21 Jackson, J. D., Yan, Y., Brunda, M. J., Kelsey, L. S. & Talmadge, J. E. Interleukin-12 enhances peripheral hematopoiesis in vivo. *Blood* **85**, 2371-2376 (1995).
22 Orange, J. S. & Biron, C. A. Characterization of early IL-12, IFN-alphabeta, and TNF effects on antiviral state and NK cell responses during murine cytomegalovirus infection. *J Immunol* **156**, 4746-4756 (1996).
23 Cousens, L. P. *et al.* Two roads diverged: interferon alpha/beta- and interleukin 12-mediated pathways in promoting T cell interferon gamma responses during viral infection. *J Exp Med* **189**, 1315-1328 (1999).
24 Le Bon, A. *et al.* Type i interferons potently enhance humoral immunity and can promote isotype switching by stimulating dendritic cells in vivo. *Immunity* **14**, 461-470 (2001).
25 Le Bon, A. *et al.* Cross-priming of CD8+ T cells stimulated by virus-induced type I interferon. *Nat Immunol* **4**, 1009-1015 (2003).
26 Mescher, M. F. *et al.* Signals required for programming effector and memory development by CD8+ T cells. *Immunol Rev* **211**, 81-92 (2006).
27 Lopez-Collazo, E., Hortelano, S., Rojas, A. & Bosca, L. Triggering of peritoneal macrophages with IFN-alpha/beta attenuates the expression of inducible nitric oxide synthase through a decrease in NF-kappaB activation. *J Immunol* **160**, 2889-2895 (1998).
28 Pacelli, R. *et al.* Nitric oxide potentiates hydrogen peroxide-induced killing of Escherichia coli. *J Exp Med* **182**, 1469-1479 (1995).
29 Xu, X. *et al.* Down-regulation of IL-12 p40 gene in Plasmodium berghei-infected mice. *J Immunol* **167**, 235-241 (2001).
30 Feng, C. *et al.* An alternate pathway for type 1 T cell differentiation. *Int Immunol* **11**, 1185-1194 (1999).
31 Sexton, A. C. *et al.* Transcriptional profiling reveals suppressed erythropoiesis, up-regulated glycolysis, and interferon-associated responses in murine malaria. *J Infect Dis* **189**, 1245-1256 (2004).
32 Schaecher, K., Kumar, S., Yadava, A., Vahey, M. & Ockenhouse, C. F. Genome-wide expression profiling in malaria infection reveals transcriptional changes

Nature Precedings : hdl:10101/npre.2011.5930.1 : Posted 27 Apr 2011

associated with lethal and nonlethal outcomes. *Infect Immun* **73**, 6091-6100 (2005).

33 Brinkmann, V., Geiger, T., Alkan, S. & Heusser, C. H. Interferon alpha increases the frequency of interferon gamma-producing human CD4+ T cells. *J Exp Med* **178**, 1655-1663 (1993).

34 Robertson, M. J. *et al.* Response of human natural killer (NK) cells to NK cell stimulatory factor (NKSF): cytolytic activity and proliferation of NK cells are differentially regulated by NKSF. *J Exp Med* **175**, 779-788 (1992).

35 Spitzer, G. & Verma, D. S. Cells with Fc gamma receptors from normal donors suppress granulocytic macrophage colony formation. *Blood* **60**, 758-766 (1982).

36 Wyler, D. J. Peripheral lymphocyte subpopulations in human falciparum malaria. *Clin Exp Immunol* **23**, 471-476 (1976).

37 Kassa, D., Petros, B., Mesele, T., Hailu, E. & Wolday, D. Characterization of peripheral blood lymphocyte subsets in patients with acute Plasmodium falciparum and P. vivax malaria infections at Wonji Sugar Estate, Ethiopia. *Clin Vaccine Immunol* **13**, 376-379 (2006).

38 Herberman, R. R., Ortaldo, J. R. & Bonnard, G. D. Augmentation by interferon of human natural and antibody-dependent cell-mediated cytotoxicity. *Nature* **277**, 221-223 (1979).

39 Kohl, S., Sigaroudinia, M., Charlebois, E. D. & Jacobson, M. A. Interleukin-12 administered in vivo decreases human NK cell cytotoxicity and antibody-dependent cellular cytotoxicity to human immunodeficiency virus-infected cells. *J Infect Dis* **174**, 1105-1108 (1996).

40 Biron, C. A., Nguyen, K. B., Pien, G. C., Cousens, L. P. & Salazar-Mather, T. P. Natural killer cells in antiviral defense: function and regulation by innate cytokines. *Annu Rev Immunol* **17**, 189-220 (1999).

41 Ordi, J. *et al.* Placental malaria is associated with cell-mediated inflammatory responses with selective absence of natural killer cells. *J Infect Dis* **183**, 1100-1107 (2001).

42 Dickensheets, H. L., Venkataraman, C., Schindler, U. & Donnelly, R. P. Interferons inhibit activation of STAT6 by interleukin 4 in human monocytes by inducing SOCS-1 gene expression. *Proc Natl Acad Sci U S A* **96**, 10800-10805 (1999).

43 Wang, X., Chen, M., Wandinger, K. P., Williams, G. & Dhib-Jalbut, S. IFN-beta-1b inhibits IL-12 production in peripheral blood mononuclear cells in an IL-10-dependent mechanism: relevance to IFN-beta-1b therapeutic effects in multiple sclerosis. *J Immunol* **165**, 548-557 (2000).

44 Kilunga Kubata, B. *et al.* Plasmodium falciparum produces prostaglandins that are pyrogenic, somnogenic, and immunosuppressive substances in humans. *J Exp Med* **188**, 1197-1202 (1998).

45 Daily, J. P. *et al.* In vivo transcriptional profiling of Plasmodium falciparum. *Malar J* **3**, 30 (2004).

46 Winkler, S. *et al.* Reciprocal regulation of Th1- and Th2-cytokine-producing T cells during clearance of parasitemia in Plasmodium falciparum malaria. *Infect Immun* **66**, 6040-6044 (1998).

Nature Precedings : hdl:10101/npre.2011.5930.1 : Posted 27 Apr 2011

## Table 1. TH1 (primarily THαβ) immunity related gene up-regulation after malaria

| U | E | E/U | A | A/U | R | R/U | Gene | TH1 Immunity |
|---|---|---|---|---|---|---|---|---|
| 0.595 | 3.656 | 6.1445378 | 1.213 | 2.0386555 | 1.003 | 1.6857143 | IFI53 | IFNa/b |
| 0.898 | 3.089 | 3.4398664 | 1.298 | 1.4454343 | 1.132 | 1.2605791 | IFI27 | |
| 0.825 | 2.375 | 2.8787879 | 1.077 | 1.3054545 | 0.928 | 1.1248485 | IFI44 | |
| 0.552 | 2.093 | 3.7916667 | 1.158 | 2.0978261 | 0.933 | 1.6902174 | G1P2 | |
| 0.51 | 2.07 | 4.0588235 | 1.031 | 2.0215686 | 0.947 | 1.8568627 | IRF1 | |
| 0.813 | 1.763 | 2.1685117 | 1.08 | 1.3284133 | 0.926 | 1.1389914 | OAS3 | |
| 0.579 | 1.829 | 3.1588946 | 1.058 | 1.8272884 | 0.977 | 1.6873921 | STAT1 | |
| 0.722 | 1.773 | 2.4556787 | 1.091 | 1.5110803 | 0.952 | 1.3185596 | IFI35 | |
| 0.881 | 1.586 | 1.800227 | 1.069 | 1.2133939 | 0.898 | 1.0192963 | OASL | |
| 0.672 | 1.498 | 2.2291667 | 1.046 | 1.5565476 | 0.954 | 1.4196429 | MX1 | |
| 0.662 | 1.532 | 2.3141994 | 1.26 | 1.9033233 | 1.097 | 1.6570997 | IRF7 | |
| 0.898 | 4.549 | 5.0657016 | 0.992 | 1.1046771 | 0.894 | 0.9955457 | IFIT4 | |
| 0.902 | 2.352 | 2.6075388 | 0.938 | 1.0399113 | 0.924 | 1.0243902 | IFIT1 | |
| 0.931 | 1.935 | 2.0784103 | 1.065 | 1.1439313 | 0.964 | 1.0354458 | IFIT2 | |
| 0.629 | 1.214 | 1.9300477 | 1.149 | 1.8267091 | 1.019 | 1.6200318 | IFITM1 | |
| 0.655 | 1.043 | 1.5923664 | 2.424 | 3.7007634 | 1.694 | 2.5862595 | IFNG | IFNg |
| 0.587 | 6.22 | 10.596252 | 1.099 | 1.8722317 | 0.823 | 1.4020443 | GBP1 | |
| 0.527 | 1.908 | 3.6204934 | 1.111 | 2.1081594 | 0.947 | 1.7969639 | GBP2 | |
| 0.755 | 1.273 | 1.6860927 | 1.182 | 1.5655629 | 0.951 | 1.2596026 | IFNGR2 | |
| 0.658 | 1.31 | 1.9908815 | 1.284 | 1.9513678 | 0.916 | 1.3920973 | IFI30 | |
| 0.781 | 1.333 | 1.7067862 | 1.322 | 1.6927017 | 1.123 | 1.4379001 | JAK2 | |
| 0.72 | 2.049 | 2.8458333 | 0.991 | 1.3763889 | 0.95 | 1.3194444 | TAP1 | |
| 0.781 | 1.371 | 1.7554417 | 1.057 | 1.3533931 | 1.067 | 1.3661972 | JAK1 | |
| 0.838 | 1.261 | 1.5047733 | 1.106 | 1.3198091 | 0.9 | 1.0739857 | FcGR2B | ADCC |
| 0.863 | 1.199 | 1.3893395 | 1.925 | 2.230591 | 1.075 | 1.2456547 | FcAR | |
| 0.514 | 0.911 | 1.7723735 | 1.658 | 3.2256809 | 1.339 | 2.6050584 | FcGR3B | |
| 0.708 | 1.208 | 1.7062147 | 1.132 | 1.5988701 | 0.896 | 1.2655367 | FcGR2A | |
| 0.64 | 4.954 | 7.740625 | 1.265 | 1.9765625 | 0.849 | 1.3265625 | FcGR1A | |
| 0.751 | 1.285 | 1.7110519 | 1.412 | 1.8801598 | 1.035 | 1.3781625 | C1QA | |
| 0.765 | 1.601 | 2.0928105 | 1.073 | 1.4026144 | 0.946 | 1.2366013 | FcGR3A | |
| 0.597 | 1.237 | 2.0720268 | 1.658 | 2.7772194 | 1.339 | 2.2428811 | FcER1G | |
| 0.953 | 0.658 | 0.6904512 | 1.298 | 1.3620147 | 1.502 | 1.5760756 | CD3z | |
| 0.819 | 0.676 | 0.8253968 | 1.455 | 1.7765568 | 1.329 | 1.6227106 | Fyn | |
| 0.656 | 1.029 | 1.5685976 | 1.219 | 1.8582317 | 1.035 | 1.5777439 | DAP12 | |
| 0.848 | 0.985 | 1.1615566 | 1.271 | 1.4988208 | 1.204 | 1.4198113 | Syk | |
| 0.566 | 0.449 | 0.7932862 | 2.044 | 3.6113074 | 1.829 | 3.2314488 | PI3K | |
| 0.748 | 1.108 | 1.4812834 | 1.152 | 1.540107 | 1.03 | 1.3770053 | Rac1 | |
| 0.83 | 1.21 | 1.4578313 | 1.25 | 1.5060241 | 1.13 | 1.3614458 | PAK1 | |
| 0.823 | 0.807 | 0.9805589 | 1.464 | 1.7788578 | 1.191 | 1.4471446 | MAP2K1 | |
| 0.721 | 0.872 | 1.2094313 | 1.516 | 2.1026352 | 1.375 | 1.9070735 | MAP2K2 | |
| 0.644 | 0.81 | 1.257764 | 1.547 | 2.4021739 | 1.382 | 2.1459627 | Arf6 | |
| 0.881 | 0.642 | 0.7287174 | 2.723 | 3.0908059 | 2.931 | 3.3269012 | KLRC3 | NK cell |
| 0.829 | 0.868 | 1.0470446 | 1.26 | 1.5199035 | 1.492 | 1.7997587 | NKEFB | |
| 0.927 | 0.848 | 0.9147789 | 1.442 | 1.5555556 | 1.432 | 1.5447681 | KIR2DL3 | |
| 0.857 | 0.694 | 0.8098016 | 2.199 | 2.5659277 | 2.1 | 2.4504084 | KIR3DL2 | |
| 0.77 | 3.235 | 4.2012987 | 0.835 | 1.0844156 | 0.798 | 1.0363636 | TNFSF10 | |
| 0.653 | 1.242 | 1.9019908 | 1.152 | 1.7641654 | 1 | 1.5313936 | Fas | |
| 0.693 | 0.991 | 1.4300144 | 1.144 | 1.6507937 | 1.084 | 1.5642136 | MICB | |
| 0.901 | 0.772 | 0.8568257 | 1.439 | 1.5971143 | 1.31 | 1.4539401 | ETS1 | |
| 0.704 | 0.899 | 1.2769886 | 1.596 | 2.2670455 | 1.379 | 1.9588068 | CEBPg | |
| 0.757 | 1.145 | 1.5125495 | 1.119 | 1.4782034 | 1.02 | 1.347424 | MEF | |
| 0.638 | 0.765 | 1.1990596 | 1.674 | 2.6238245 | 1.458 | 2.2852665 | ID2 | |
| 0.71 | 1.397 | 1.9676056 | 1.092 | 1.5380282 | 0.884 | 1.2450704 | IL15 | |
| 0.908 | 0.738 | 0.8127753 | 1.521 | 1.6751101 | 1.626 | 1.7907489 | GZMM | |
| 0.768 | 0.962 | 1.2526042 | 1.568 | 2.0416667 | 1.199 | 1.5611979 | CD58 | |

Nature Precedings : hdl:10101/npre.2011.5930.1 : Posted 27 Apr 2011

Nature Precedings : hdl:10101/npre.2011.5930.1 : Posted 27 Apr 2011

| | | | | | | | | |
|---|---|---|---|---|---|---|---|---|
| 0.548 | 1.223 | 2.2317518 | 1.491 | 2.7208029 | 0.963 | 1.7572993 | CCR1 | TH1 Chemokine |
| 0.381 | 0.974 | 2.5564304 | 2.085 | 5.4724409 | 1.713 | 4.496063 | CCL3 | |
| 0.689 | 0.735 | 1.0667634 | 3.206 | 4.6531205 | 2.65 | 3.8461538 | CCL4 | |
| 0.986 | 5.194 | 5.2677485 | 1.045 | 1.0598377 | 0.956 | 0.969574 | CXCL10 | |
| 1.029 | 1.576 | 1.5315841 | 0.835 | 0.8114674 | 0.83 | 0.8066084 | CXCL9 | |
| 0.824 | 1.265 | 1.5351942 | 0.924 | 1.1213592 | 0.808 | 0.9805825 | CXCL11 | |
| 0.863 | 0.641 | 0.7427578 | 1.495 | 1.7323291 | 1.557 | 1.8041715 | GZMK | CD8 T cell |
| 0.795 | 0.651 | 0.8188679 | 1.98 | 2.490566 | 1.739 | 2.1874214 | GZMB | |
| 0.881 | 0.638 | 0.7241771 | 1.236 | 1.4029512 | 1.522 | 1.7275823 | GZMA | |
| 0.829 | 0.765 | 0.9227986 | 1.432 | 1.7273824 | 1.385 | 1.6706876 | RUNX3 | |
| 0.656 | 1.07 | 1.6310976 | 1.308 | 1.9939024 | 1.106 | 1.6859756 | Rel | |
| 0.925 | 0.812 | 0.8778378 | 1.497 | 1.6183784 | 1.593 | 1.7221622 | CD3g | |
| 0.871 | 0.892 | 1.0241102 | 2.021 | 2.3203215 | 1.719 | 1.9735936 | CDC42 | |
| 0.749 | 0.692 | 0.9238985 | 1.885 | 2.5166889 | 1.524 | 2.034713 | Kras | |
| 0.78 | 1.159 | 1.4858974 | 1.209 | 1.55 | 1.094 | 1.4025641 | ICOS | |
| 0.784 | 0.915 | 1.1670918 | 1.497 | 1.9094388 | 1.486 | 1.8954082 | CD28 | |
| 0.77 | 1.007 | 1.3077922 | 1.224 | 1.5896104 | 1.038 | 1.3480519 | Bcl10 | |
| 0.816 | 0.87 | 1.0661765 | 1.557 | 1.9080882 | 1.45 | 1.7769608 | MALT1 | |

**Table 2. TH1 (primary TH12) immunity un-changed after malaria**

| | | | | | | | | |
|---|---|---|---|---|---|---|---|---|
| 0.755 | 1.273 | 1.6860927 | 1.182 | 1.5655629 | 0.951 | 1.2596026 | IFNGR2 | Macrophag e |
| 1.02 | 0.91 | 0.8921569 | 1.16 | 1.1372549 | 1.13 | 1.1078431 | CD40L | Activation |
| 0.804 | 1.271 | 1.5808458 | 1.132 | 1.4079602 | 1.098 | 1.3656716 | TNFalpha | |
| 0.97 | 1.04 | 1.0721649 | 1.01 | 1.0412371 | 1.03 | 1.0618557 | TNFbeta | |
| 0.733 | 1.263 | 1.7230559 | 1.293 | 1.7639836 | 0.864 | 1.1787176 | TLR2 | |
| 0.744 | 1.125 | 1.5120968 | 1.385 | 1.8615591 | 0.991 | 1.3319892 | TLR4 | |
| 0.739 | 1.051 | 1.4221922 | 1.408 | 1.9052774 | 1.044 | 1.4127199 | TLR8 | |
| 0.99 | 1.04 | 1.0505051 | 1.03 | 1.040404 | 1 | 1.010101 | IL12A | |
| 1.02 | 1.04 | 1.0196078 | 0.96 | 0.9411765 | 0.95 | 0.9313725 | IL12B | |
| 1.13 | 1.08 | 0.9557522 | 0.96 | 0.8495575 | 0.94 | 0.8318584 | IL12RB1 | |
| 1.04 | 1.16 | 1.1153846 | 1.02 | 0.9807692 | 0.97 | 0.9326923 | IL12RB2 | |
| 1.04 | 0.78 | 0.75 | 1.22 | 1.1730769 | 1.27 | 1.2211538 | STAT4 | |
| 1.03 | 1.08 | 1.0485437 | 1.02 | 0.9902913 | 1.01 | 0.9805825 | iNOS | |
| 1.054 | 1 | 0.9487666 | 1 | 0.9487666 | 1.04 | 0.9867173 | ASS | |
| 1 | 1.27 | 1.27 | 0.94 | 0.94 | 0.96 | 0.96 | MMP9 | |
| 0.87 | 0.98 | 1.1264368 | 1.19 | 1.3678161 | 1.12 | 1.2873563 | TRAF6 | |
| 0.98 | 1.03 | 1.0510204 | 0.99 | 1.0102041 | 1 | 1.0204082 | TRANCE | |
| 1.05 | 1.06 | 1.0095238 | 0.94 | 0.8952381 | 0.94 | 0.8952381 | RANK | |
| 0.99 | 1.039 | 1.0494949 | 0.97 | 0.979798 | 0.99 | 1 | ERM | |
| 1.07 | 1.08 | 1.0093458 | 0.93 | 0.8691589 | 0.92 | 0.8598131 | M-CSF | Macrophag e |
| 1.02 | 1.03 | 1.0098039 | 0.97 | 0.9509804 | 0.97 | 0.9509804 | GM-CSF | Proliferation |
| 1.11 | 1.03 | 0.9279279 | 0.94 | 0.8468468 | 0.89 | 0.8018018 | IL-3 | |
| 0.92 | 1.04 | 1.1304348 | 1.13 | 1.2282609 | 1.04 | 1.1304348 | RUNX1 | |
| 0.86 | 0.89 | 1.0348837 | 0.85 | 0.9883721 | 0.84 | 0.9767442 | Myb | |
| 1.21 | 1.25 | 1.0330579 | 0.81 | 0.6694215 | 0.87 | 0.7190083 | Myc | |
| 1 | 1.24 | 1.24 | 1 | 1 | 0.95 | 0.95 | CEBPalpha | |
| 1.04 | 1.04 | 1 | 1 | 0.9615385 | 1.03 | 0.9903846 | HOXB7 | |
| 1.06 | 1.17 | 1.1037736 | 0.95 | 0.8962264 | 0.97 | 0.9150943 | GATA1 | |
| 1.07 | 1.06 | 0.9906542 | 0.95 | 0.8878505 | 0.98 | 0.9158879 | GATA2 | |
| 1.12 | 1.19 | 1.0625 | 0.82 | 0.7321429 | 0.86 | 0.7678571 | Tal1 | |
| 0.358 | 0.952 | 2.6592179 | 2.495 | 6.9692737 | 1.351 | 3.773743 | MafB | |
| 0.841 | 0.809 | 0.9619501 | 1.401 | 1.665874 | 1.295 | 1.5398335 | TGFBR2 | Macrophag e |
| 0.85 | 1.355 | 1.5941176 | 1.024 | 1.2047059 | 0.727 | 0.8552941 | CD36 | Inhibition |
| 0.706 | 0.972 | 1.3767705 | 1.307 | 1.8512748 | 1.209 | 1.7124646 | CD47 | |
| 0.943 | 0.783 | 0.8303287 | 1.521 | 1.6129374 | 1.097 | 1.1633086 | CD163 | |
| 0.522 | 0.781 | 1.4961686 | 1.776 | 3.4022989 | 1.556 | 2.9808429 | JunB | |

| | | | | | | | |
|---|---|---|---|---|---|---|---|
| 0.51 | 2.07 | 4.0588235 | 1.031 | 2.0215686 | 0.947 | 1.8568627 | IRF1 |
| 0.73 | 0.95 | 1.3013699 | 2.62 | 3.5890411 | 1.81 | 2.4794521 | HMOX1 |
| 0.83 | 1.24 | 1.4939759 | 1.27 | 1.5301205 | 0.97 | 1.1686747 | SIRPalpha |
| 0.7 | 1.319 | 1.8842857 | 1.48 | 2.1142857 | 1.02 | 1.4571429 | PU.1 |

**Red color means greater than 2-fold change;**
**yellow color means 1.5-2-fold change;**
**green color means no change**

Nature Precedings : hdl:10101/npre.2011.5930.1 : Posted 27 Apr 2011

Nature Precedings : hdl:10101/npre.2011.5930.1 : Posted 27 Apr 2011

**Table 3. TH17 related gene up-regulation after malaria**

| U | E | E/U | A | A/U | R | R/U | Gene | TH17Immunity |
|---|---|---|---|---|---|---|---|---|
| 0.767 | 0.775 | 1.0104302 | 1.275 | 1.6623207 | 1.382 | 1.8018253 | IL32 | IL17 CD4 Tcell |
| 0.343 | 0.779 | 2.271137 | 2.922 | 8.5189504 | 1.389 | 4.0495627 | IL8 | |
| 0.805 | 1.265 | 1.5714286 | 2.276 | 2.8273292 | 1.931 | 2.3987578 | IL1B | |
| 0.804 | 1.271 | 1.5808458 | 1.132 | 1.4079602 | 1.098 | 1.3656716 | TNFalpha | |
| 0.653 | 1.019 | 1.56049 | 1.346 | 2.0612557 | 1.156 | 1.770291 | TGFB1 | |
| 0.841 | 0.809 | 0.9619501 | 1.401 | 1.665874 | 1.295 | 1.5398335 | TGFBR2 | |
| 0.795 | 0.907 | 1.1408805 | 1.849 | 2.3257862 | 1.491 | 1.8754717 | OSM | |
| 0.77 | 1.333 | 1.7311688 | 1.04 | 1.3506494 | 0.865 | 1.1233766 | STAT3 | |
| 0.764 | 0.894 | 1.1701571 | 2.98 | 3.9005236 | 1.648 | 2.1570681 | CEBPD | |
| 0.459 | 0.991 | 2.1590414 | 1.694 | 3.6906318 | 1.163 | 2.5337691 | CEBPB | |
| 0.903 | 1.552 | 1.7187154 | 0.899 | 0.9955703 | 0.787 | 0.8715393 | S100A8 | Neutrophil |
| 0.707 | 1.568 | 2.2178218 | 1.049 | 1.4837341 | 0.852 | 1.2050919 | S100A9 | |
| 0.667 | 0.97 | 1.4542729 | 2.827 | 4.2383808 | 1.699 | 2.5472264 | CXCL2 | |
| 0.923 | 1.027 | 1.1126761 | 2.027 | 2.1960997 | 1.333 | 1.4442037 | CXCL3 | |
| 0.745 | 1.4 | 1.8791946 | 1.182 | 1.5865772 | 0.946 | 1.2697987 | CD63 | |
| 0.708 | 1.26 | 1.779661 | 1.183 | 1.670904 | 0.917 | 1.2951977 | CD157 | |
| 0.722 | 1.948 | 2.6980609 | 1.155 | 1.599723 | 0.923 | 1.2783934 | ICAM1 | |
| 0.725 | 0.911 | 1.2565517 | 1.167 | 1.6096552 | 1.054 | 1.4537931 | ITGB2 | |
| 0.954 | 2.945 | 3.0870021 | 0.939 | 0.9842767 | 0.801 | 0.8396226 | NCF1 | |
| 0.827 | 1.471 | 1.7787183 | 1.116 | 1.3494559 | 0.953 | 1.1523579 | NCF4 | |
| 0.742 | 1.047 | 1.4110512 | 1.202 | 1.6199461 | 1.011 | 1.3625337 | CD97 | |
| 0.709 | 1.382 | 1.9492243 | 1.16 | 1.6361072 | 0.801 | 1.1297602 | FPR1 | |
| 0.918 | 1.131 | 1.2320261 | 1.619 | 1.7636166 | 1.23 | 1.3398693 | Pentraxin3 | |
| 0.723 | 1.08 | 1.4937759 | 2.135 | 2.9529737 | 1.895 | 2.6210235 | PGES2 | |
| 0.635 | 1.087 | 1.711811 | 2.079 | 3.2740157 | 1.218 | 1.9181102 | C3AR1 | Complement |
| 0.623 | 0.908 | 1.4574639 | 2.628 | 4.2182986 | 1.571 | 2.5216693 | C5R1 | |
| 0.552 | 0.881 | 1.5960145 | 2.297 | 4.1612319 | 1.392 | 2.5217391 | DAF | |
| 0.751 | 1.285 | 1.7110519 | 1.412 | 1.8801598 | 1.035 | 1.3781625 | C1QA | |
| 0.67 | 0.914 | 1.3641791 | 1.349 | 2.0134328 | 1.166 | 1.7402985 | FactorD | |
| 0.761 | 1.093 | 1.4362681 | 1.418 | 1.8633377 | 1.121 | 1.4730618 | Properdin | |

**Red color means greater than 2-fold change;**
**yellow color means 1.5-2-fold change;**
**green color means no change**

Nature Precedings : hdl:10101/npre.2011.5930.1 : Posted 27 Apr 2011

**Table 4. TH2 related gene un-changed after malaria**

| U | E | E/U | A | A/U | R | R/U | Gene | TH2Immunity |
|---|---|---|---|---|---|---|---|---|
| 1.09 | 1.06 | 0.9724771 | 0.97 | 0.8899083 | 1.02 | 0.9357798 | IL4 | IL4 CD4 Tcell |
| 1.07 | 1.08 | 1.0093458 | 0.97 | 0.9065421 | 1 | 0.9345794 | IL4R | |
| 1.02 | 1.08 | 1.0588235 | 0.99 | 0.9705882 | 0.97 | 0.9509804 | IL5 | |
| 1.06 | 1 | 0.9433962 | 0.98 | 0.9245283 | 0.99 | 0.9339623 | IL5R | |
| 1.08 | 1.1 | 1.0185185 | 0.95 | 0.8796296 | 0.92 | 0.8518519 | IL9 | |
| 1.08 | 1.08 | 1 | 0.99 | 0.9166667 | 0.95 | 0.8796296 | IL9R | |
| 0.94 | 0.99 | 1.0531915 | 1.15 | 1.2234043 | 1.09 | 1.1595745 | IL10 | |
| 0.93 | 0.85 | 0.9139785 | 1.08 | 1.1612903 | 1.17 | 1.2580645 | IL10RA | |
| 1.09 | 1.05 | 0.9633028 | 0.95 | 0.8715596 | 0.92 | 0.8440367 | IL13 | |
| 0.98 | 1.14 | 1.1632653 | 1.23 | 1.255102 | 0.99 | 1.0102041 | IL13RA1 | |
| 1.13 | 1.01 | 0.8938053 | 1.01 | 0.8938053 | 1.01 | 0.8938053 | IL13RA2 | |
| 1.1 | 1.13 | 1.0272727 | 0.91 | 0.8272727 | 0.96 | 0.8727273 | CCR3 | |
| 1.08 | 0.96 | 0.8888889 | 0.98 | 0.9074074 | 1.01 | 0.9351852 | CCR4 | |
| 1.11 | 1.2 | 1.0810811 | 0.86 | 0.7747748 | 0.95 | 0.8558559 | CCR8 | |
| 1.08 | 1.12 | 1.037037 | 0.89 | 0.8240741 | 0.94 | 0.8703704 | CCL17 | |
| 0.99 | 1.1 | 1.1111111 | 0.91 | 0.9191919 | 0.95 | 0.959596 | CCL22 | |
| 1.09 | 1.04 | 0.9541284 | 0.92 | 0.8440367 | 0.93 | 0.853211 | CRTH2 | |
| 1.05 | 0.94 | 0.8952381 | 1.08 | 1.0285714 | 0.98 | 0.9333333 | CD30 | |
| 0.92 | 0.98 | 1.0652174 | 1.13 | 1.2282609 | 1.17 | 1.2717391 | OX40 | |
| 1.32 | 1.31 | 0.9924242 | 0.93 | 0.7045455 | 0.89 | 0.6742424 | OX40L | |
| 1.01 | 0.88 | 0.8712871 | 1.01 | 1 | 0.99 | 0.980198 | STAT6 | |
| 1 | 0.92 | 0.92 | 1.18 | 1.18 | 1.16 | 1.16 | GATA3 | |
| 0.98 | 1 | 1.0204082 | 0.96 | 0.9795918 | 1 | 1.0204082 | NFATc | |
| 0.92 | 0.91 | 0.9891304 | 1.27 | 1.3804348 | 1.31 | 1.423913 | Gfi-1 | |
| 0.96 | 1.09 | 1.1354167 | 1.1 | 1.1458333 | 1.07 | 1.1145833 | SPHK1 | |
| 1.05 | 1.02 | 0.9714286 | 0.97 | 0.9238095 | 1 | 0.952381 | SH2D1A | |
| 1.12 | 1.08 | 0.9642857 | 0.87 | 0.7767857 | 0.85 | 0.7589286 | ST2L | |
| 1.11 | 1.06 | 0.954955 | 0.94 | 0.8468468 | 0.98 | 0.8828829 | A3AR | Mast cell |
| 1.1 | 1.13 | 1.0272727 | 0.91 | 0.8272727 | 0.96 | 0.8727273 | CCR3 | |
| 1.03 | 1.22 | 1.184466 | 0.89 | 0.8640777 | 0.99 | 0.961165 | FCER2 | |
| 1.14 | 0.63 | 0.5526316 | 1.17 | 1.0263158 | 1.72 | 1.5087719 | FCER1A | |
| 1.09 | 1.13 | 1.0366972 | 0.93 | 0.853211 | 1.01 | 0.9266055 | LTC4S | |
| 0.95 | 0.96 | 1.0105263 | 1.11 | 1.1684211 | 1.05 | 1.1052632 | MGST2 | |
| 0.89 | 0.93 | 1.0449438 | 1.19 | 1.3370787 | 1.13 | 1.2696629 | MGST3 | |
| 0.92 | 0.92 | 1 | 0.84 | 0.9130435 | 0.85 | 0.923913 | CysLTR1 | |
| 1.07 | 1.04 | 0.9719626 | 0.94 | 0.8785047 | 0.94 | 0.8785047 | CysLTR2 | |
| 1.02 | 1.08 | 1.0588235 | 0.99 | 0.9705882 | 0.97 | 0.9509804 | IL5 | Eosinophil |
| 1.11 | 1.01 | 0.9099099 | 0.97 | 0.8738739 | 1.02 | 0.9189189 | Eotaxin1 | |
| 1.18 | 1.17 | 0.9915254 | 0.88 | 0.7457627 | 0.89 | 0.7542373 | Eotaxin2 | |

**Red color means greater than 2-fold change;**
**yellow color means 1.5-2-fold change;**
**green color means no change**
**blue color means down-regulation**

**Table 5. Correlation between Fc receptor/complement receptor and STAT1/STAT4 in acute febrile malaria**

| Gene | STAT1 | STAT4 |
|---|---|---|
| FCER1G | 0.9 (0.001) | -0.71 (0.01) |
| FCGR1A | 0.88 (0.001) | -0.74 (0.01) |
| FCGR2A | 0.79 (0.01) | -0.79 (0.01) |
| FCGR2B | 0.74 (0.01) | -0.77 (0.01) |
| Fas | 0.71 (0.01) | -0.64 (0.05) |
| C3AR | 0.86 (0.001) | -0.73 (0.01) |
| DAF | 0.64 (0.05) | -0.66 (0.02) |
| C1QA | 0.71 (0.01) | -0.64 (0.05) |
| Properdin | 0.82 (0.01) | -0.68 (0.02) |

Nature Precedings : hdl:10101/npre.2011.5930.1 : Posted 27 Apr 2011

Nature Precedings : hdl:10101/npre.2011.5930.1 : Posted 27 Apr 2011

**Figure Legends**

**FIG. 1.** IL-10 related gene up-regulation during acute febrile malaria; Comparison of chemokine levels between microarray analysis and serum plasma

**FIG. 2**. Gene-to-gene correlation.

Panel A: negative correlation between STAT1 and STAT4,

Pearson's correlation coefficient (r) and its P value (p) were calculated for each graph

**FIG. 3**.

3-A IFN-αβ centered pathway analysis. Network analysis was performed by using IFN-α and IFN-β as the central nodes. We used software to select IFN-α or IFN-β related genes from our genelist. 24 IFN-α or IFN-β related genes were selected. Red entities mean proteins. Green entities mean small molecules including phospholipids and drugs. Hexon means groups of family proteins. A---B means A binds to B to form a complex. A-→ B means A binds B's promoter region to up-regulate B. A--+->B means A induces B release. A---|B means A causes B's degradation or inhibition. Little square in the middle of the line means there is a third molecule which regulates the conversion from A to B.

3-B IL-4 centered pathway analysis. Network analysis was performed by using IL-4 as the central nodes. We used software to select IL-4 related genes from our genelist. Only 4 IL-4 related genes were selected.

3-C IL-12 centered pathway analysis. Network analysis was performed by using IL-12 as the central nodes. We used software to select IL-12 related genes from our genelist. Only 4 IL-12A/IL12B related genes were selected.

3-D TGF-β/Oncostatin M centered pathway analysis. Network analysis was performed by using TGF-β/Oncostatin M as the central nodes. We used software to select TGF-

β/Oncostatin M related genes from our genelist. 22 TGF-β/Oncostatin M related genes were selected. Red entities mean s proteins.

**FIG. 4.** Proposed model for the four types of host immunity.

Green color means un-changed and red color means up-regulated genes after malaria infection. TH2 immunity is induced by STAT6 activated by IL-4. THαβ immunity is induced by STAT1 & STAT3α activated by IFN-αβ. TH1 immunity is induced by STAT4 activated by IL-12. TH17 immunity is induced by STAT3β activated by TGF-β/IL-6 to induce downstream PMN related genes and proinflammatory cytokines (TNFα, IL1, IL8). In THαβ immunity, IFN-α/β can suppress STAT6. IFN-α/β also suppress iNOS and IL-12 via IL-10 dependent mechanism. Up-regulated STAT1 can suppress GATA3 and STAT4. STAT1 can up-regulate IRF1, T-bet, IgG Fc receptors, Fas, C3a receptor. IRF1 induced TRAIL, T-bet induced NK cell killer receptors, and Fas can enhance NK cell cytotoxicity. Up-regulated IgG Fc receptors can mediate ADCC. In T cell, T-bet can up-regulate IFN-γ. Up-regulated IFN-γ can activate STAT1 signaling via IFN-γ receptor. However, the IFN-γ receptor is suppressed during lymphocyte maturation. Thus, THαβ immunity can be separated from TH1 immunity.

**FIG 5.**

5-A. Summary of gene expression of the four immunological pathways during the early malaria, acute febrile malaria, and remission period compared to un-infection baseline. Red color means gene expression with greater than two-fold up-regulation. Yellow color means gene expression with 1.5 to 2-fold up-regulation. Green color means gene expression was not changed. Square shape means genes which activate the cell, and ellipse shape means genes which suppress the cell. Although interferon alpha/beta was

Nature Precedings : hdl:10101/npre.2011.5930.1 : Posted 27 Apr 2011

not detected, ( IRF7) means that this up-regulated gene regulates the transcription of interferon alpha/beta.

5-B. Gene expression during early malaria infection,

5-C. Gene expression during acute febrile malaria infection,

5-D. Gene expression during the remission period

Nature Precedings : hdl:10101/npre.2011.5930.1 : Posted 27 Apr 2011

**FIG 1.**

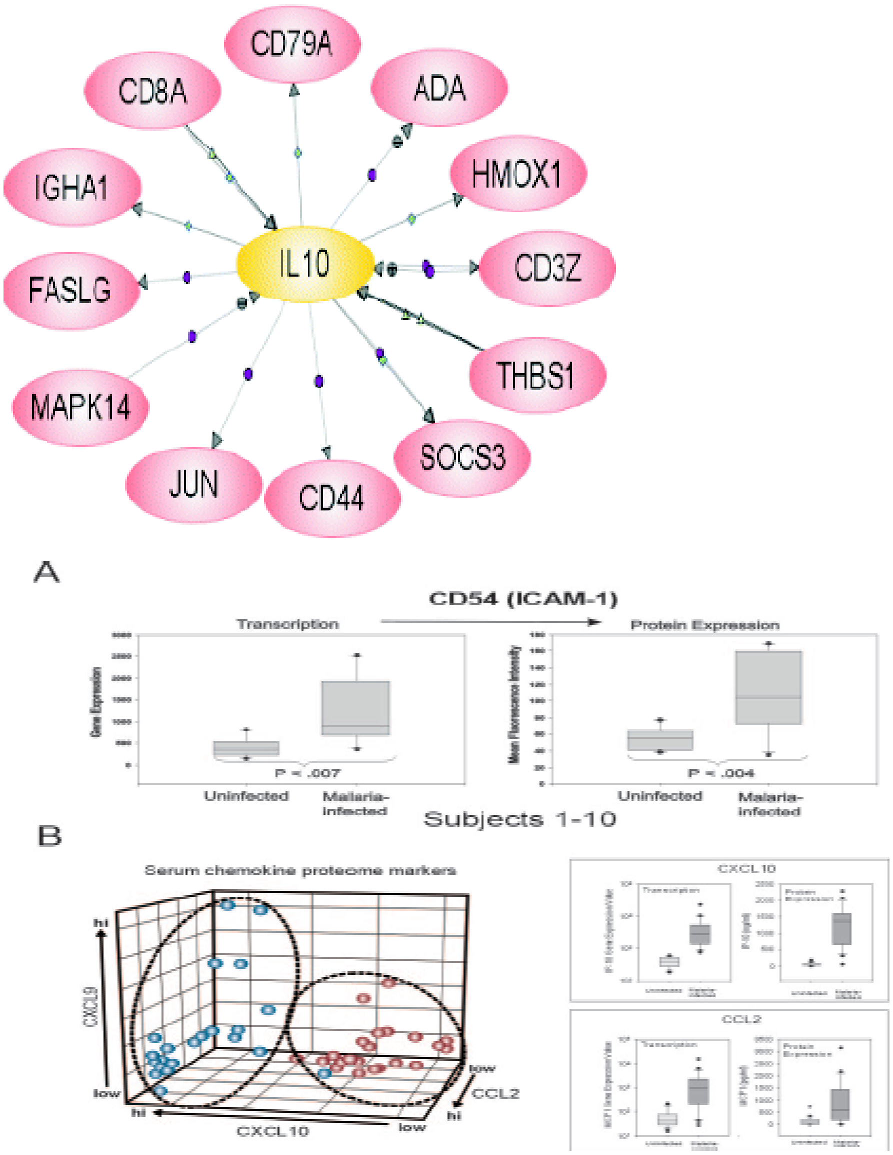

(cited from Ockenhouse & Hu et al.(2006) Infect Immun 74(10),5561)

Nature Precedings : hdl:10101/npre.2011.5930.1 : Posted 27 Apr 2011

**FIG. 2.**

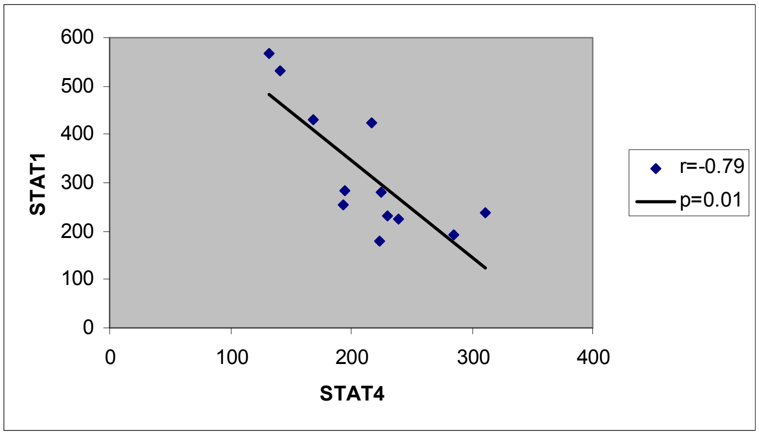

**Panel A**

Nature Precedings : hdl:10101/npre.2011.5930.1 : Posted 27 Apr 2011

**FIG. 3.**

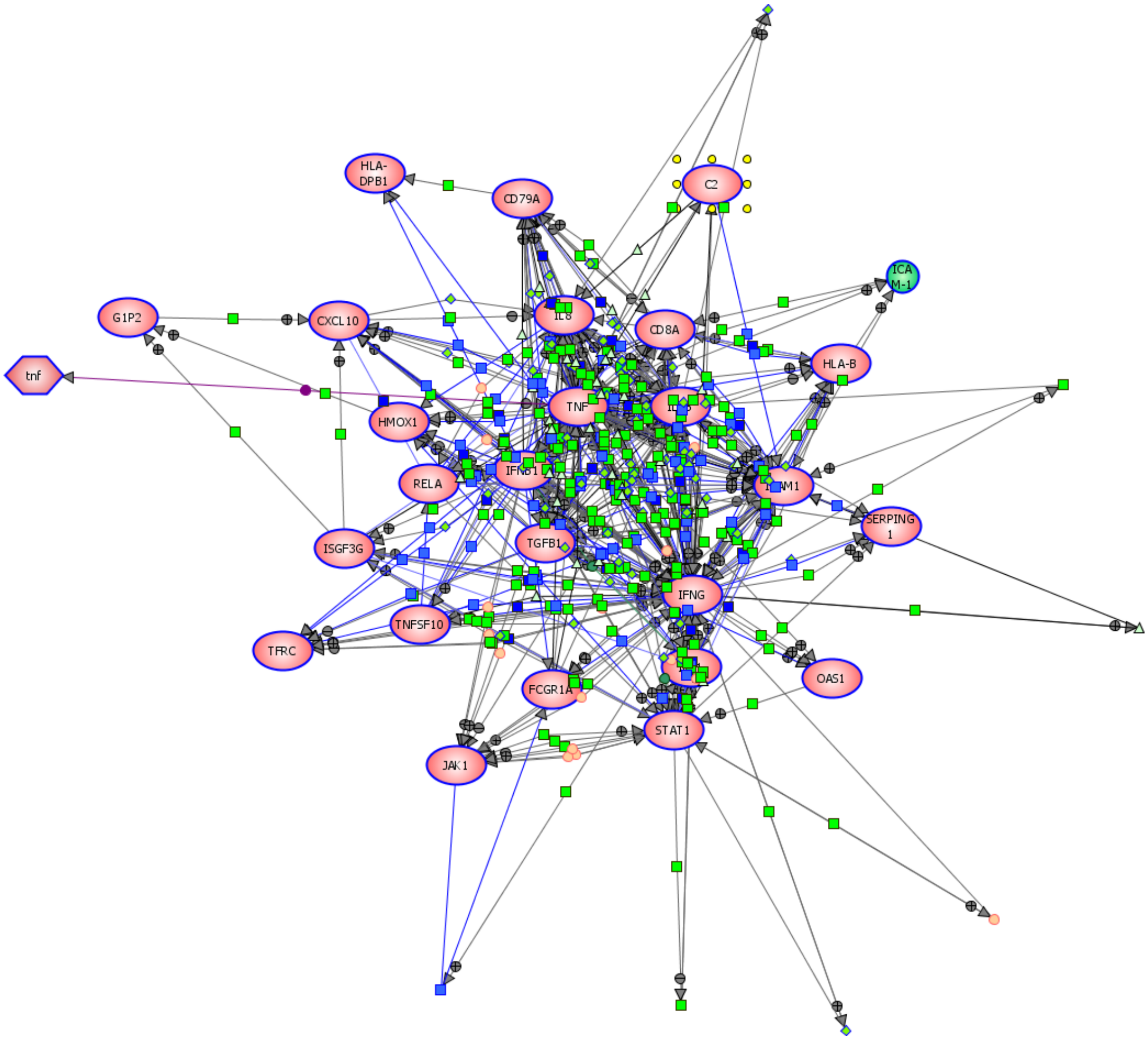

**3-A**

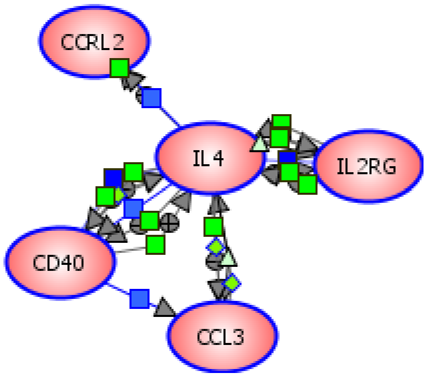

**3-B**

Nature Precedings : hdl:10101/npre.2011.5930.1 : Posted 27 Apr 2011

Nature Precedings : hdl:10101/npre.2011.5930.1 : Posted 27 Apr 2011

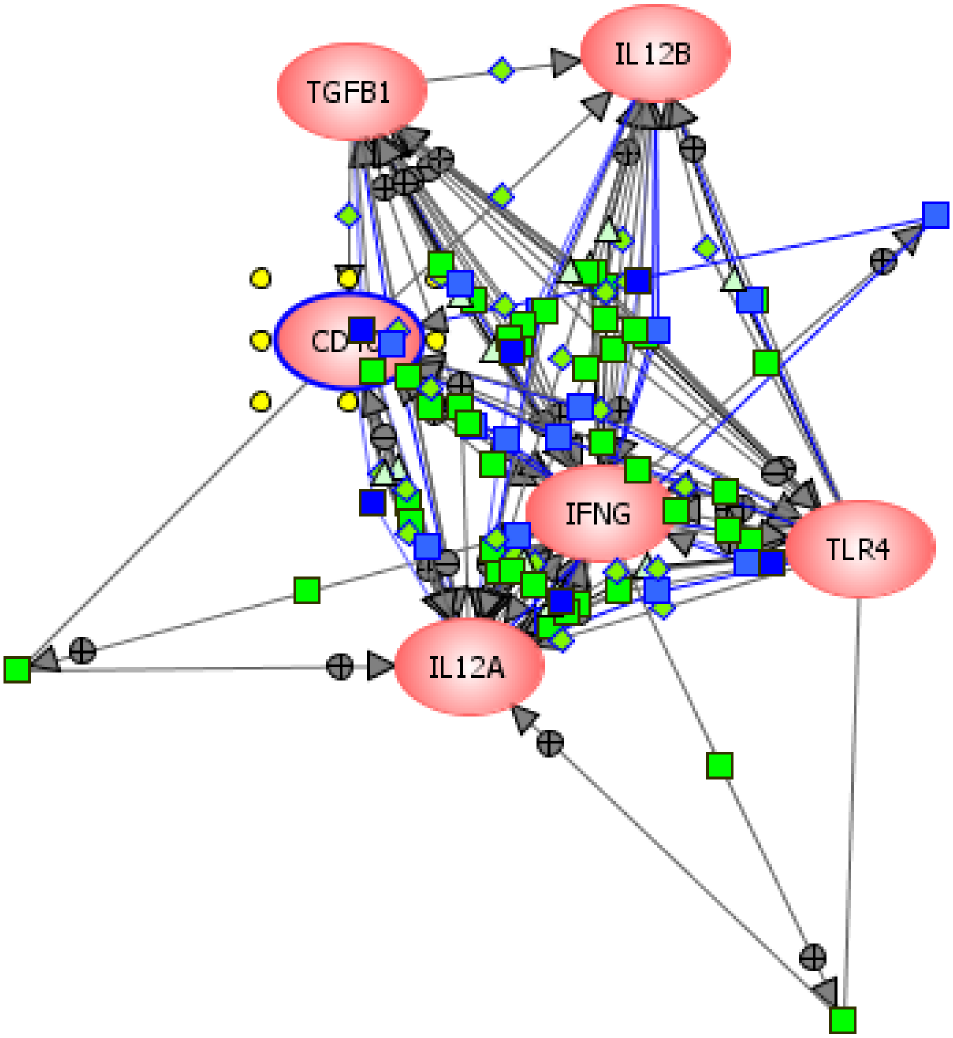

**3-C**

Nature Precedings : hdl:10101/npre.2011.5930.1 : Posted 27 Apr 2011

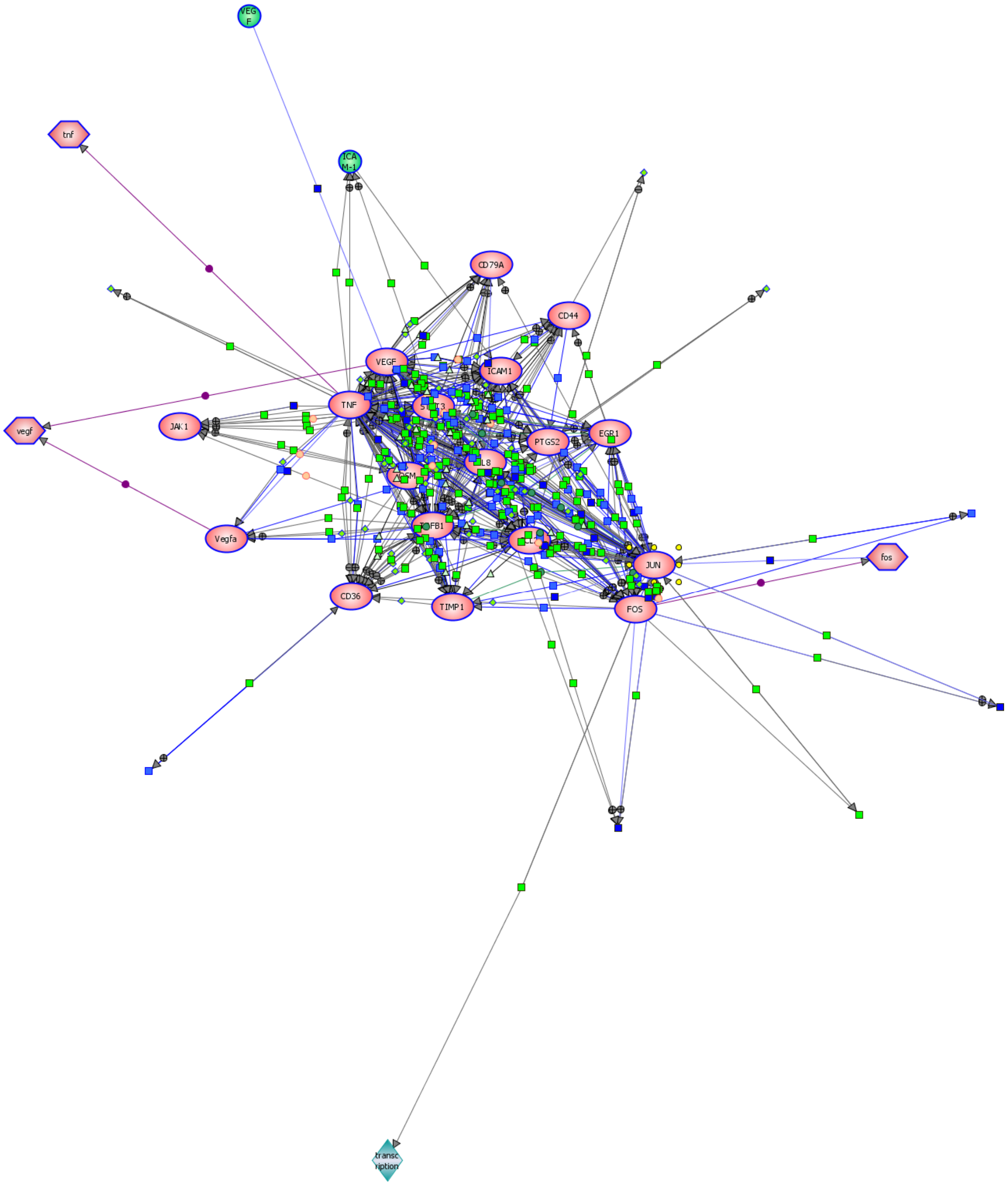

**3-D**

**FIG. 4.**

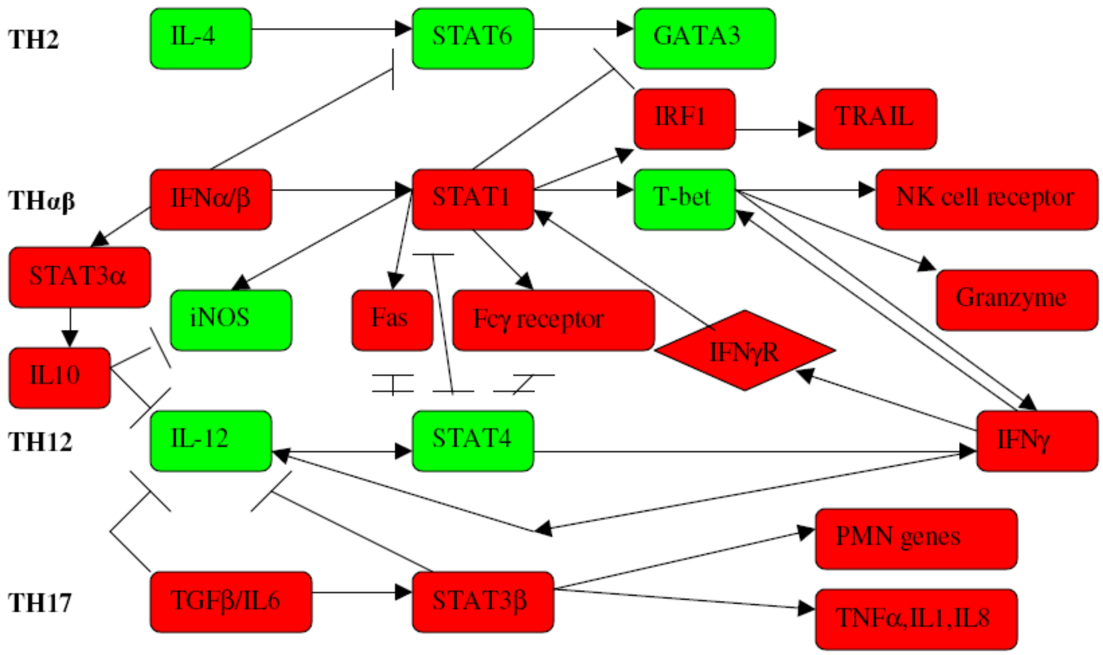

*Traditional TH1 includes THαβ & TH12 immunity
Green: un-changed; Red: up-regulation

Nature Precedings : hdl:10101/npre.2011.5930.1 : Posted 27 Apr 2011

Nature Precedings : hdl:10101/npre.2011.5930.1 : Posted 27 Apr 2011

**FIG. 5.**

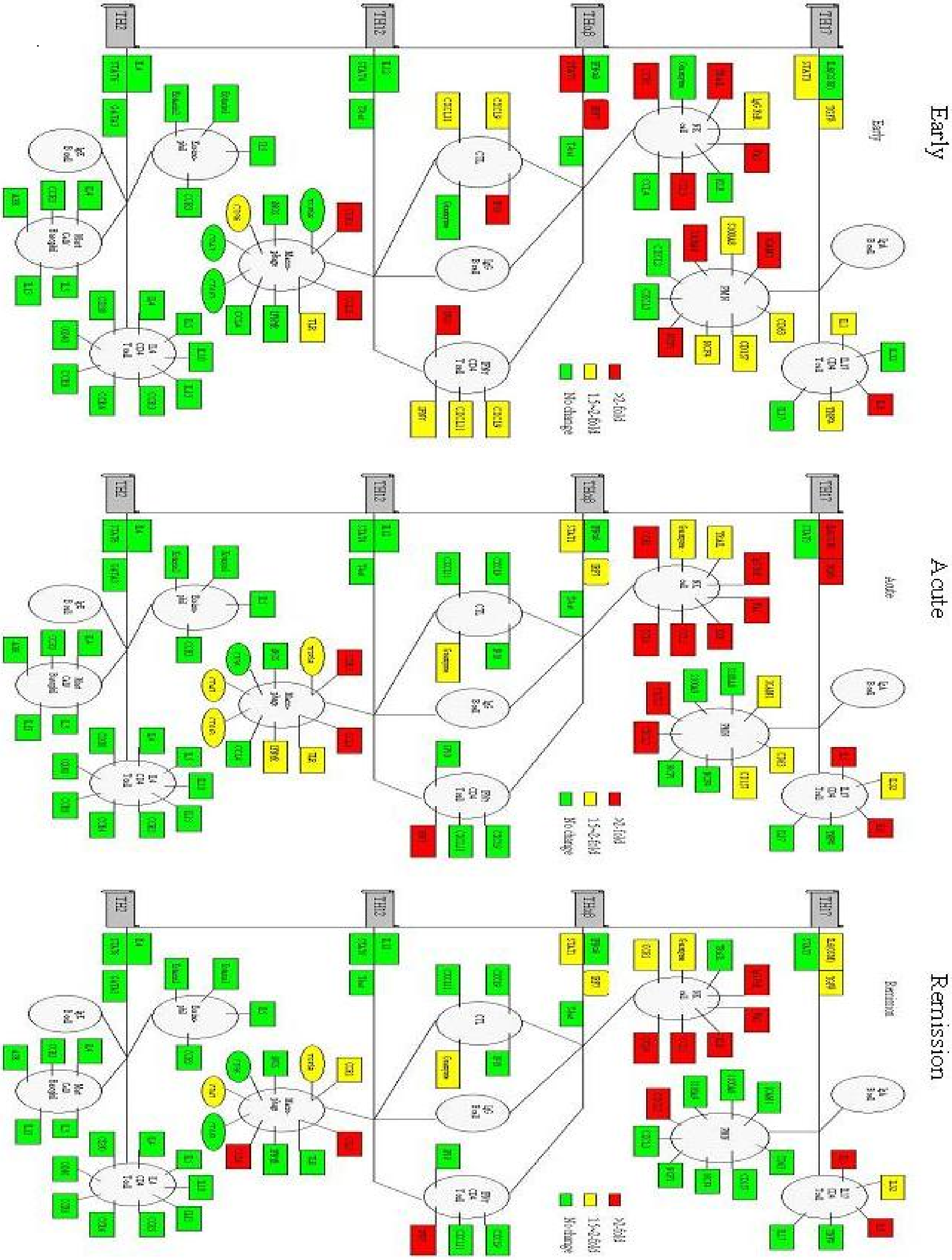

**5-A**

Nature Precedings : hdl:10101/npre.2011.5930.1 : Posted 27 Apr 2011

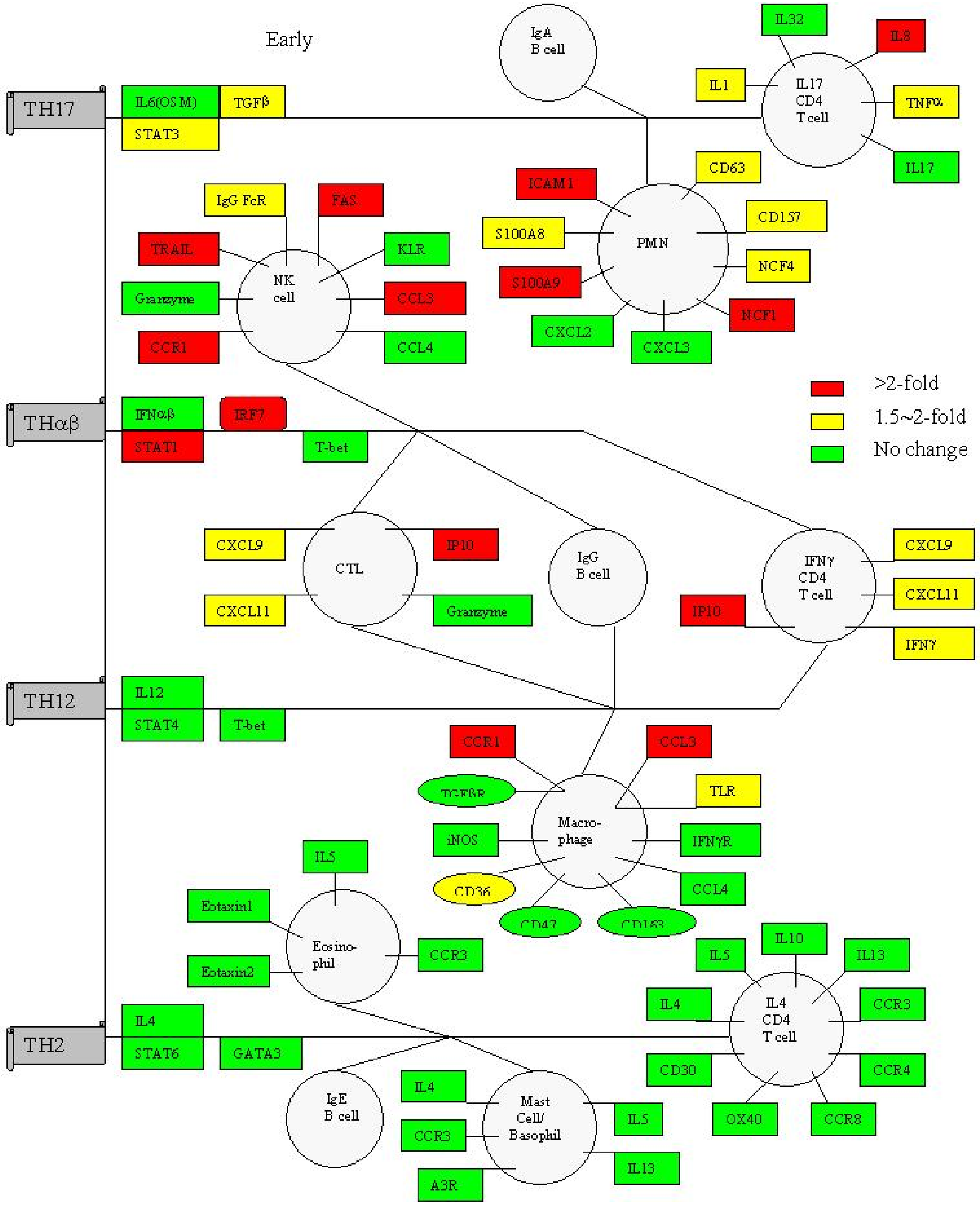

**5-B**

Nature Precedings : hdl:10101/npre.2011.5930.1 : Posted 27 Apr 2011

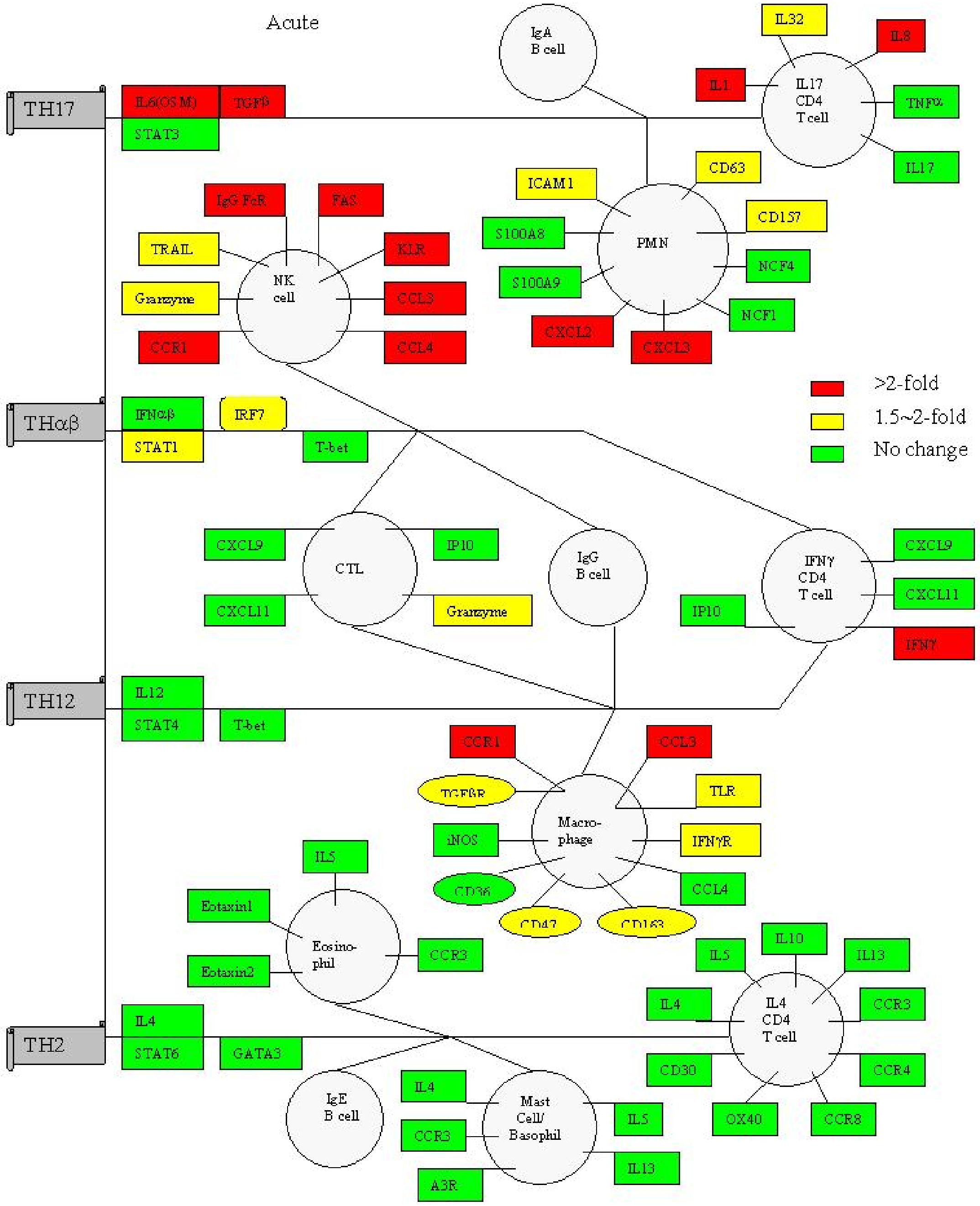

**5-C**

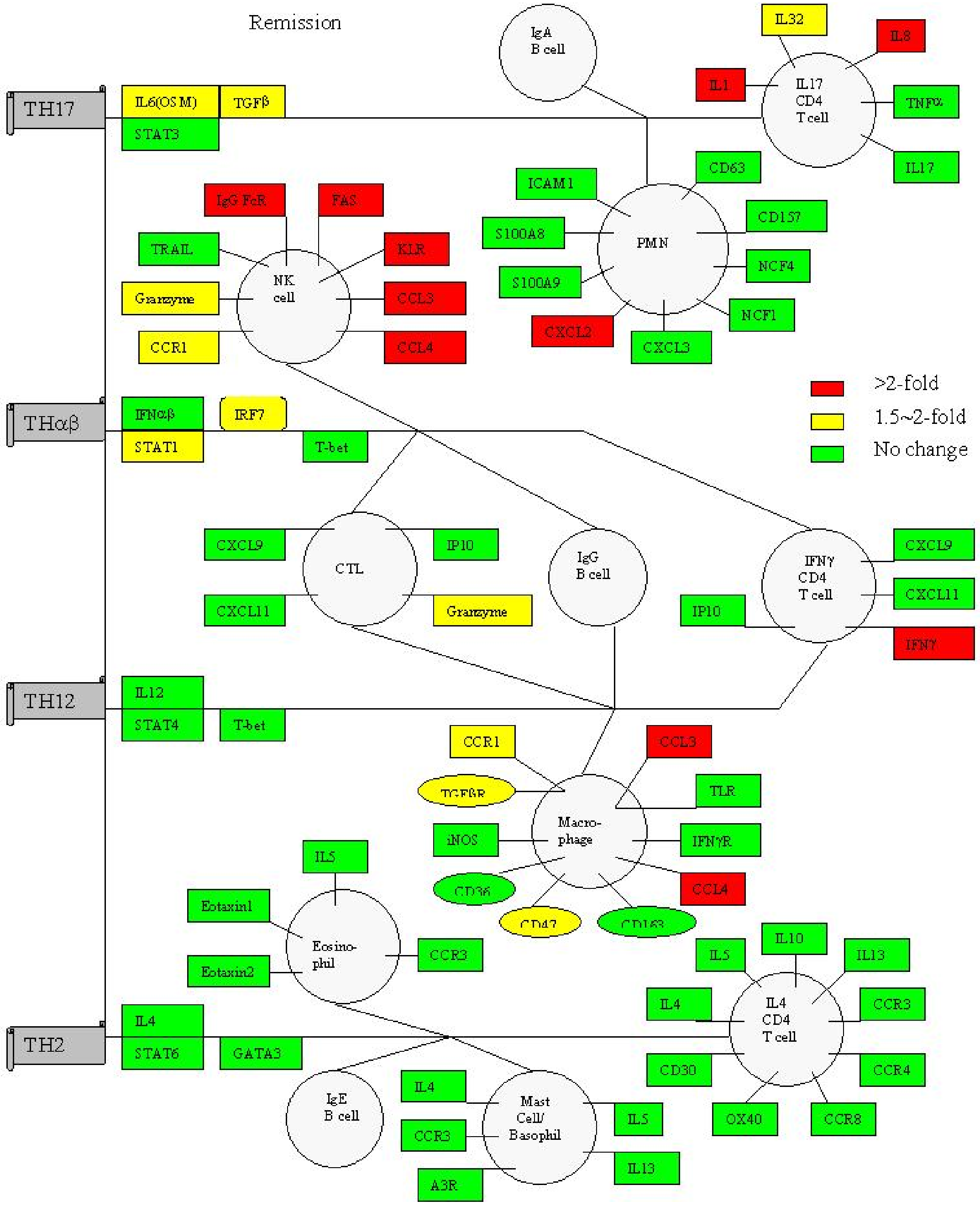

**5-D**

Nature Precedings : hdl:10101/npre.2011.5930.1 : Posted 27 Apr 2011
